# HepaClear, a blood-based panel combining novel methylated CpG sites and protein markers, for the detection of early-stage hepatocellular carcinoma

**DOI:** 10.1186/s13148-023-01508-7

**Published:** 2023-06-12

**Authors:** Yi Bai, Juan Xu, Deqiang Li, Xiaoyu Zhang, Dapeng Chen, Fucun Xie, Longmei Huang, Xiaotian Yu, Haitao Zhao, Yamin Zhang

**Affiliations:** 1 Department of Hepatobiliary Surgery, Tianjin First Central Hospital, School of Medicine, Nankai University, Tianjin, China; 2 Department of Liver Surgery, State Key Laboratory of Complex Severe and Rare Diseases, Peking Union Medical College Hospital, Chinese Academy of Medical Sciences and Peking Union Medical College, Beijing, China; 3 Department of Infectious Diseases, Central Hospital of Shengli Oilfield, Dongying, China; 4 Hangzhou New Horizon Health Technology Co., Ltd, Hangzhou, China; 5 The First Central Clinical School, Tianjin Medical University, Tianjin, China; 6 Department of Thoracic Surgery, National Cancer Center/National Clinical Research Center for Cancer/Cancer Hospital, Chinese Academy of Medical Sciences and Peking Union Medical College, Beijing, China; 7 State Key Laboratory of Molecular Oncology, National Cancer Center/National Clinical Research Center for Cancer/Cancer Hospital, Chinese Academy of Medical Sciences and Peking Union Medical College, Beijing, China; 8 Central Laboratory, National Cancer Center/National Clinical Research Center for Cancer/Cancer Hospital and Shenzhen Hospital, Chinese Academy of Medical Sciences and Peking Union Medical College, Shenzhen, China

**Keywords:** DNA methylation, Hepatocellular carcinoma, 850K methylation array, Multi-target panel, Early detection

## Abstract

**Background:**

Early screening and detection of hepatocellular carcinoma (HCC) can efficiently improve patient prognosis. We aimed to identify a series of hypermethylated DNA markers and develop a blood-based HCC diagnosis panel containing DNA methylation sites and protein markers with improved sensitivity for early-stage HCC detection.

**Results:**

Overall, 850K methylation arrays were performed using paired tissue DNA samples from 60 HCC patients. Ten candidate hypermethylated CpG sites were selected for further evaluation by quantitative methylation-specific PCR with 60 pairs of tissue samples. Six methylated CpG sites, along with α-fetoprotein (AFP) and des-gamma-carboxyprothrombin (DCP), were assayed in 150 plasma samples. Finally, an HCC diagnosis panel, named HepaClear, was developed in a cohort consisting of 296 plasma samples and validated in an independent cohort consisting of 198 plasma samples. The HepaClear panel, containing 3 hypermethylated CpG sites (cg14263942, cg12701184, and cg14570307) and 2 protein markers (AFP and DCP), yielded a sensitivity of 82.6% and a specificity of 96.2% in the training set and a sensitivity of 84.7% and a specificity of 92.0% in the validation set. The HepaClear panel had higher sensitivity (72.0%) for early-stage HCC than AFP (≥ 20 ng/mL, 48.0%) and DCP (≥ 40 mAU/mL, 62.0%) and detected 67.5% of AFP-negative HCC patients (AFP ≤ 20 ng/mL).

**Conclusions:**

We developed a multimarker HCC detection panel (HepaClear) that shows high sensitivity for early-stage HCC. The HepaClear panel exhibits high potential for HCC screening and diagnosis from an at-risk population.

**Supplementary Information:**

The online version contains supplementary material available at 10.1186/s13148-023-01508-7.

## Background

Liver cancer is the third leading cause of cancer death worldwide [1] and creates a significant public health burden. Hepatocellular carcinoma (HCC) accounts for up to 85–90% of liver cancers [2]. In China, HCC is the second most deadly cancer [3], and the 5-year survival rate of HCC patients is only approximately 14% [4], while over 50% of early-stage HCC (BCLC 0/A) patients can live more than 5 years after standard treatment [5]. A poor early detection rate (less than 50%) and a lack of effective therapies for advanced-stage HCC cause poor prognosis in HCC patients [6]. Therefore, the screening and diagnosis of early-stage HCC in high-risk populations are essential for improving the overall survival rate and reducing treatment costs [7]. According to The Asian Pacific Association for the Study of the Liver (APASL) HCC guidelines, the major risk factors for HCC in China include chronic hepatitis B virus (HBV) infection and liver cirrhosis (LC). Hence, patients with the abovementioned risk factors are recommended for HCC surveillance [8].

Currently, early detection of HCC or monitoring of HCC recurrence mainly relies on ultrasonography (US), serum alpha-fetoprotein (AFP) levels and tissue biopsy [9]. However, these methods show limitations in diagnostic accuracy and sensitivity for early-stage HCC (BCLC 0/A), including approximately 50% sensitivity for AFP and 45% for US alone, even though the sensitivity of combining AFC and US is only 63% [10–12]. For protein markers, although a combination of AFP, des-gamma-carboxyprothrombin (DCP) and lectin-bound AFP (AFP-L3) has higher sensitivity and accuracy than AFP alone [13, 14], neither DCP nor AFP-L3 can improve the performance in distinguishing HBV-associated early HCC and LC with chronic hepatitis B (CHB) [15]. Therefore, it is critical to find novel biomarkers to detect early-stage HCC in high-risk populations.

Cell-free DNA (cfDNA), released from apoptotic, necrotic, and living cells, has been widely used as a plasma-based biomarker in cancer screening and diagnosis [16]. Circulating tumor DNA (ctDNA), a small portion of cfDNA derived from tumor cells, contains genetic defects identical to the tumor cells from which they originated. It has been reported that cfDNA plays an important role in the detection of early-stage HCC [17, 18]. Among the different mechanisms underlying genetic/epi-genetic alterations, DNA methylation changes have been reported to contribute to tumorigenesis [19]. Aberrant DNA methylation can occur at the precancerous lesion or early stage and has a strong correlation with metastasis and recurrence [20]. Moreover, abnormal DNA methylation patterns usually cause up/downregulated gene expression, resulting in silencing of tumor suppressor genes involved in hepatocarcinogenesis [21]. Therefore, aberrant methylation signals can be useful targets for cancer surveillance and treatment.

Currently, cfDNA methylation biomarkers have been applied in the evaluation of HCC diagnosis and prognosis, and different blood-based methylation panels have shown high sensitivity and specificity in clinical practice [22–24]. However, the limited tissue sample number and traditional 450K Methylation BeadChip array used in previous studies may have led to bias and missed some potential markers. In recent studies, almost all HCC patients and normal individuals included were from the United States, and these patients had different genetic backgrounds and major HCC etiologies from the Chinese population [25]. The leading cause of HCC in China is chronic hepatitis B virus (HBV) infection [26], while in the United States is chronic hepatitis C virus (HCV) infection. The carcinogenic mechanism of HBV and HCV is different. HBV can integrate into the host genome and cause the aberrant activation or suppression of the nearby gene [27]. In contrast, HCV disturbs cell growth and apoptosis through some specific proteins, such as HCV core protein and HCV non-structual protein NS3 [28]. As it reported by Lambert et al. [29], the different mechanisms lead to distinct methylation profiles of HBV-related HCC and HCV-related HCC. Therefore, it is essential to identify more HCC methylation biomarkers applicable to Chinese HCC patients. Here, we conducted an HCC-specific DNA methylation biomarker screening and verification study and then selected appropriate hypermethylated DNA markers and protein markers to build and validate a multitarget panel named HepaClear. These results show the potential of the HepaClear panel for clinical HCC screening and diagnosis in the Chinese population with high HCC risk.

## Results

### Aberrant methylation signatures in 60 paired HCC tissue samples

To screen for genes that contain hypermethylated CpGs in HCC samples, we evaluated genome-wide methylation profiles between 60 pairs of tumor and adjacent tissues by 850K Methylation BeadChip array. The clinical and pathological features of 60 HCC patients are summarized in Additional file 1: Table S1. Etiologically, 41 patients (68.3%) were HBV-positive, and 46 patients (76.7%) had cirrhosis, partly due to the high rate of HBV infection and liver cirrhosis among Chinese HCC patients. For screening early-stage HCC methylation biomarkers, we enrolled 73.4% of HCC patients had early-stage tumors, and 58.3% had low AFP levels (< 20 ng/mL).

**Fig. 1:**
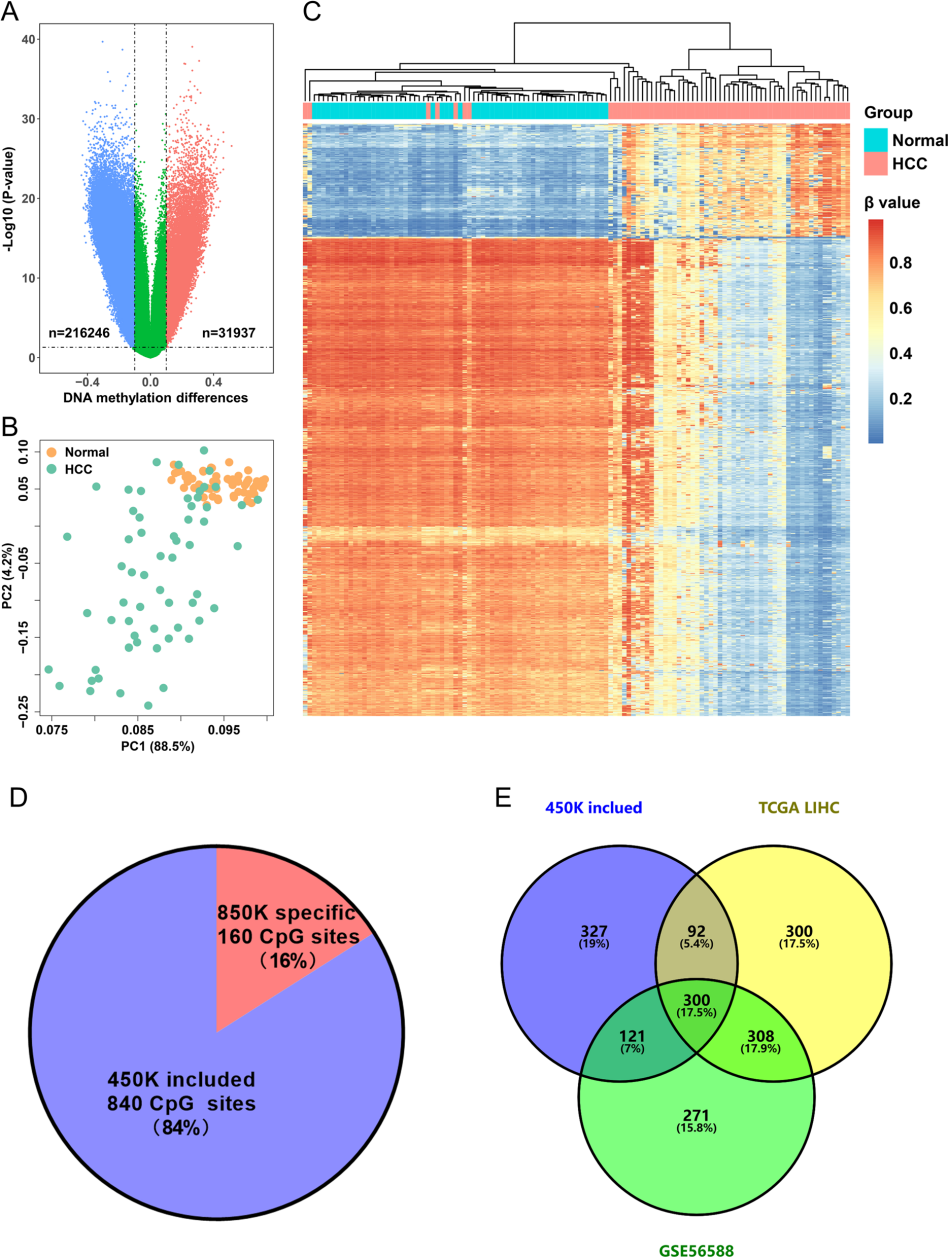
Differential methylation between HCC and normal liver tissues detected by 850K Methylation BeadChip array. **A** The volcano plot shows 31,937 hypermethylated CpG sites (red) and 216,246 hypomethylated sites (blue). The x-axis represents the DNA methylation differences, while the y-axis represents the *p value*. **B** Principle component plot of HCC and normal methylation based on beta value. **C** Unsupervised hierarchical clustering of the top 1000 methylated CpG sites. The rows represent different CpG sites, and the columns represent different tissue samples. The color in the heatmap represents the methylation beta values. **D** Hypermethylation locus distribution, whether included in 450K or not. **E** Venn diagram of the hypermethylation markers identified in this study (n = 840, 450K included), in TCGA-LIHC (n = 1000) and in GSE56588 (n = 1000)

We found that ~ 38.7% of targets were significantly altered (*p* < 0.05, |∆β|> 0.1), while only 3.7% were hypermethylated (Fig. 1A). Principal component analysis (PCA) revealed that normal tissues clustered with one another, whereas HCC tumor tissues were more dispersed, indicating greater heterogeneity in methylation signatures in HCC (Fig. 1B). The top 1000 ranked targets based on |Δβ| could efficiently distinguish HCC and normal tissues (Fig. 1C). Gene ontology (GO) analyses showed enrichment in terms related to keratinization and positive chemotaxis (Additional file 1: Fig. S1A). We also found enrichment in pathways involved in tight junction, cardiomyopathy, and inflammatory mediator regulation (Additional file 1: Fig. S1B).

From 31,937 significantly hypermethylated target CpG sites, we selected the top 1000 sites with the highest |Δβ| (Additional file 1: Table S2) and found that 16% of them were not included in the 450K Methylation BeadChip array (Fig. 1D). These methylated sites may have the potential to become biological markers, which the traditional 450K Methylation BeadChip array cannot detect because of technical limitations. Among the other 840 target sites included in the 450K Methylation BeadChip array, 300 overlapped with both the top 1000 hypermethylated sites in the TCGA-LIHC dataset and the top 1000 sites in the GSE56588 dataset, indicating good consistency of our study with previous studies (Fig. 1E).

### Selection of the markers used in the HCC diagnostic model

To reduce false-positive cases caused by partially methylated CpG sites from normal tissue-derived cfDNA in plasma, we sought to identify potential hypermethylation HCC markers, which show little or no apparent methylation in adjacent normal tissues. From the top 1000 sites, we identified 132 sites with Δβ greater than 0.3 and an average β less than 0.1 in adjacent tissues (Additional file 1: Table S3). We further integrated these sites with ROC curve analysis data from 60 pairs of tissues and selected 32 sites with AUC values higher than 0.85 and YIs no less than 0.80 (Additional file 1: Table S4). All 32 sites showed good performance in distinguishing HCC and normal tissues (Additional file 1: Fig. S2). The top 10 methylated CpG sites with the highest ∆β were chosen for further tissue validation assays. The UCSC RefGenes of the 10 methylated CpG sites are cyclin-dependent kinase-like 2 (CDC2-related kinase) (*CDKL2*), ubiquitin specific peptidase 44 (*USP44*), zinc finger family member 783 (*ZNF783*), forkhead box E3 (*FOXE3*), methylene-tetrahydrofolate dehydrogenase 2 (*MTHFD2*), cyclin-dependent kinase inhibitor 2A (*CDKN2A*), lysyl oxidase-like 3 (*LOXL3*), TLR4 interactor with leucine-rich repeats (*TRIL*), and chromosome 5 open reading frame 49 (*C5orf49*). In addition, cg20172627 is located within the intergenic region (Chr2.25439110) (Additional file 1: Table S4).

**Fig. 2:**
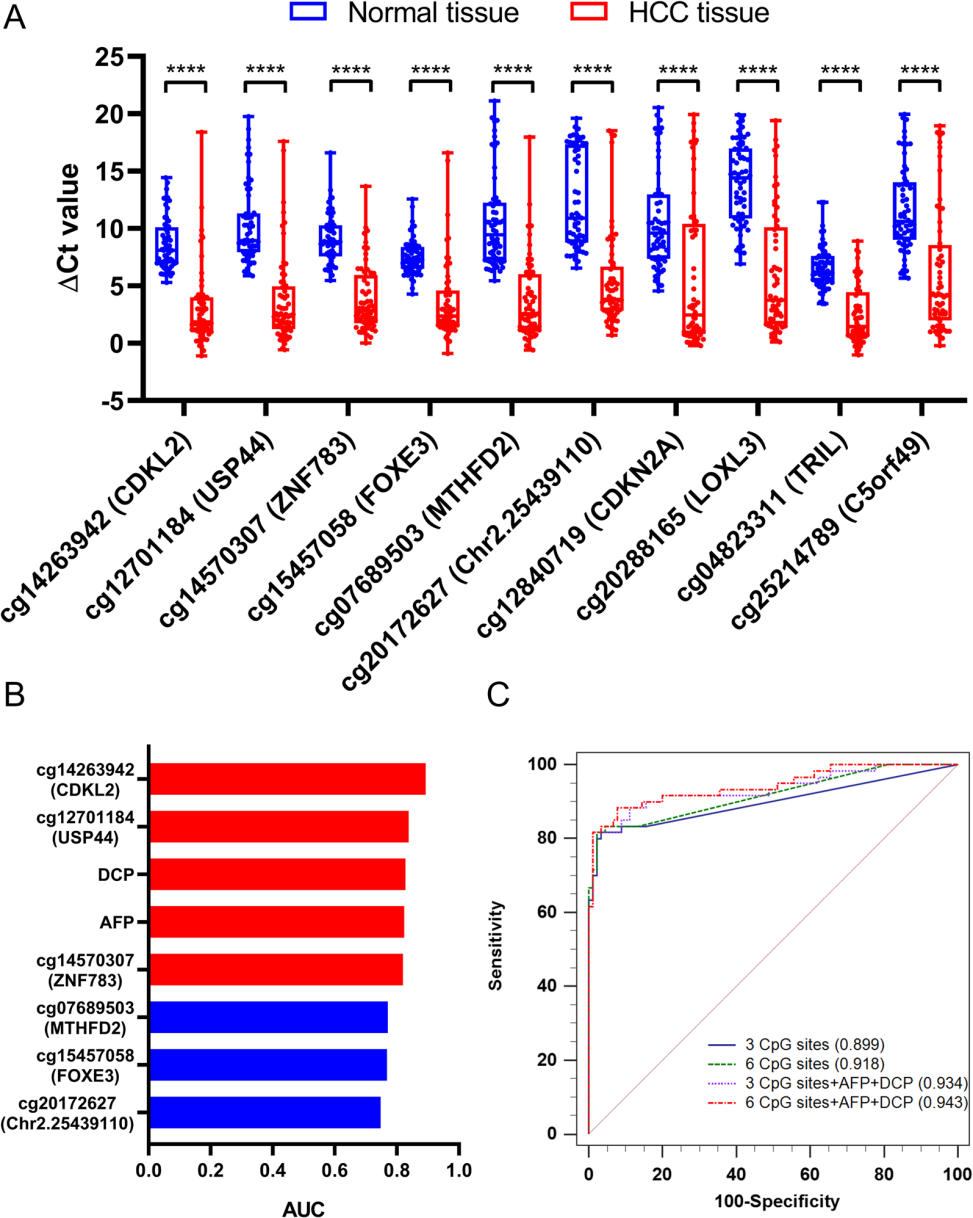
Identification of biomarkers for the HCC diagnostic model development by qMSP. **A** Methylation levels of 10 candidate methylated CpG sites as quantified by qMSP in 60 pairs of HCC and normal tissues. The y-axis represents methylation levels (ΔCt = Ct_reference_ = Ct_target_), in which a lower value represents a relatively higher methylation level. ****p < 0.0001. **B** Area under the ROC curve (AUC) of eight biomarkers in diagnosing HCC from normal plasma samples. Markers that have AUCs of > 0.8 and < 0.8 are indicated in red and blue, respectively. **C** ROC curves for 4 different biomarker panels in the plasma pilot study. The 4 panels were as follows: 3 methylated CpG sites (cg14263942, cg12701184, and cg14570307), 3 methylated CpG sites with AFP and DCP, 6 methylated CpG sites (cg14263942, cg12701184, cg14570307, cg15457058, cg07689503, and cg20172627), and 6 methylated CpG sites with AFP and DCP. The AUC values for each curve are included in parentheses

The 10 hypermethylated CpG sites were tested by TaqMan qMSP in 60 pairs of HCC and adjacent normal tissues, along with leukocytes and two HCC cell lines. All ten sites showed significantly higher methylation in HCC tissues than in normal tissues (Fig. 2A), and nine of them showed high methylation in both HepG2 and Huh7 cells (Additional file 1: Fig. S3). As a result of ROC curve analysis, AUC values of the ten marker genes ranged from 0.750 to 0.915 among 60 pairs of tissue samples, while YI varied from 0.617 to 0.850 (Table 1). Six of the ten sites (cg14263942, cg12701184, cg14570307, cg15457058, cg07689503, and cg20172627) showed a sensitivity of 78.3–85.0% and a specificity of 96.7–100%.

**Table 1:** Distinguishing performance of 10 hypermethylation markers in tissue validation set

Marker region (Gene)	AUC (95% CI)	Cut-off ΔCt value (Ct_tar_-Ct_ref_)	Sensitivity (%)	Specificity (%)	Youden index
cg14263942(CDKL2)	0.914 (0.848–0.957)	4.99	85.0	100.0	0.850
cg12701184(USP44)	0.907 (0.841–0.953)	5.35	80.0	100.0	0.800
cg14570307(ZNF783)	0.914 (0.849–0.958)	6.22	80.0	96.7	0.767
cg15457058(FOXE3)	0.879 (0.807–0.932)	5.38	81.7	96.7	0.783
cg07689503(MTHFD2)	0.915 (0.850–0.958)	6.03	78.3	98.3	0.767
cg20172627(Chr2.25439110)	0.891 (0.822–0.941)	6.99	78.3	96.7	0.750
cg12840719(CDKN2A)	0.750 (0.663–0.824)	3.8	61.7	100.0	0.617
cg20288165(LOXL3)	0.870 (0.796–0.924)	6.62	68.3	100.0	0.683
cg04823311(TRIL)	0.887 (0.816–0.937)	3.24	73.3	100.0	0.733
cg25214789(C5orf49)	0.809 (0.728–0.875)	5.27	61.7	100.0	0.617

### Determining the marker combination for the HCC diagnostic model

The abovementioned 6 hypermethylated CpG sites were selected for further verification in a pilot study of 150 plasma samples. Two protein markers, AFP and DCP, which have relatively high clinical utility for the diagnosis of HCC in Chinese patients [30] were also included in the biomarker assay. Clinical characteristics from 150 participants are listed in Additional file 1: Table S5. All 8 markers exhibited significantly increased methylation or protein levels in the HCC group (Additional file 1: Fig. S4A). Three methylated CpG sites (cg14263942, cg12701184, and cg14570307) and two protein markers had AUC values of over 0.80 (Fig. 2B), and each of the three methylated CpG sites detected ≥ 65% HCCs with a specificity of > 93% (Additional file 1: Fig. S4B).

Furthermore, we applied a stepwise logistic regression analysis with backward marker elimination to determine the optimized marker combination. The performance of diagnostic models built from different marker groups is shown in Additional file 1: Table S6. Since the model performance dropped little after eliminating cg15457058, cg07689503 and/or cg20172627 (Fig. 2C, Additional file 1: Table S6), the remaining 3 hypermethylated CpG sites, combined with AFP and DCP, were finally included for multitarget HCC diagnostic model training and verification. These markers were included to develop the diagnostic model named HepaClear.

To evaluate the limit of detection (LOD) of qMSP reaction system for the three methylation biomarkers, we used positive control (PC) and negative control (NC) composed of different concentrations of Huh7 cell DNA containing methylated target sequences. The quadruplex qMSP assay could fully detect three hypermethylated CpG sites with a minimum of 50 pg Huh7 cell DNA in 0.5% PC (Additional file 1: Figure S5, Table S7).

### Construction and validation of the HepaClear panel

In total, 494 participants were recruited to participate further in the plasma biomarker study. Plasma samples from 296 participants were used for diagnostic model construction, and samples from 198 participants were used for model validation. The general clinical characteristics of the study population are shown in Table 2. Among HCC cases, 55% in the training set and 51% in the validation set were diagnosed at BCLC stage 0 and A, while patients with relatively low AFP levels (< 20 ng/mL) accounted for 47.8% and 40.8% in the two sets.

**Table 2:** Characteristics of study participants in the training and validation sets

		Training set									Validation set				
		HCC (n = 138)	LC (n = 59)		CHB (n = 59)		Healthy (n = 40)		*P* value*		HCC (n = 98)	LC (n = 40)	CHB (n = 30)	Healthy (n = 30)	*P* value*
*Age (year)*															
Median (IQR)		56 (50–64)	50 (43–56)		45 (37–54)		42 (34–54)		< 0.001		57 (51–64)	51 (43–57)	50 (35–56)	48 (35–55)	< 0.001
*Gender*															
Male (%)	113 (79.7)		45 (76.3)	30 (50.8)		25 (62.5)		< 0.001		82 (83.7)		30 (75.0)	21 (70.0)	17 (56.7)	0.02
Female (%)	25 (20.3)		14 (23.7)	29 (49.2)		15 (37.5)				16 (16.3)		10 (25.0)	9 (30.0)	13 (43.3)	
*Etiology*						NA		0.016						NA	0.507
HBV (%)	129 (93.5)		48 (81.4)	59 (100)						88 (89.8)		35 (87.5)	30 (100)		
Alcohol (%)	2 (1.4)		4 (6.8)	0						3 (3.1)		2 (5.0)	0		
NAFLD (%)	2 (1.4)		2 (3.4)	0						2 (2)		0	0		
Other (%)	5 (3.7)		5 (8.4)	0						5 (5.1)		3 (7.5)	0		
*Cirrhosis (%)*	123 (89.1)		59 (100)	NA		NA				90 (91.8)		40 (100)	NA	NA	
*AFP (ng/mL)*															
Median (IQR)	28.9 (4.8–822.7)		4.2 (2.7–7.6)	4.1 (2.4–6.1)		2.0 (1.3–3.2)		< 0.001		54.9 (3.5–992.9)		4.4 (2.9–9.4)	4.3 (3.1–7.1)	3.6 (3.0–4.3)	< 0.001
< 20 (%)	66 (47.8)		53 (89.8)	56 (94.9)		40 (100)		< 0.001		40 (40.8)		36 (90.0)	28 (93.3)	30 (100)	< 0.001
20–400 (%)	31 (22.5)		6 (10.2)	3 (5.1)		0				30 (30.6)		4 (10.0)	2 (6.7)	0	
> 400 (%)	41 (29.7)		0	0		0				28 (28.6)		0	0	0	
*BCLC stage*			NA	NA		NA		NA				NA	NA	NA	NA
0 (%)	6 (4.3)									6 (6.1)					
A (%)	70 (50.7)									44 (44.9)					
B (%)	27 (19.6)									14 (14.3)					
C (%)	27 (19.6)									31 (31.6)					
D (%)	8 (5.8)									3 (3.1)					

*AFP* a-fetoprotein, *BCLC* Barcelona Clinic Liver Cancer, *CHB* chronic hepatitis B, *LC* liver cirrhosis, *HBV* hepatitis B virus, *HCC* hepatocellular carcinoma, *IQR* interquartile range, *NAFLD* nonalcoholic fatty liver disease

*Statistical tests performed: Chi-square test of independence, Kruskal–Wallis test, and Wilcoxon rank-sum test

**Fig. 3:**
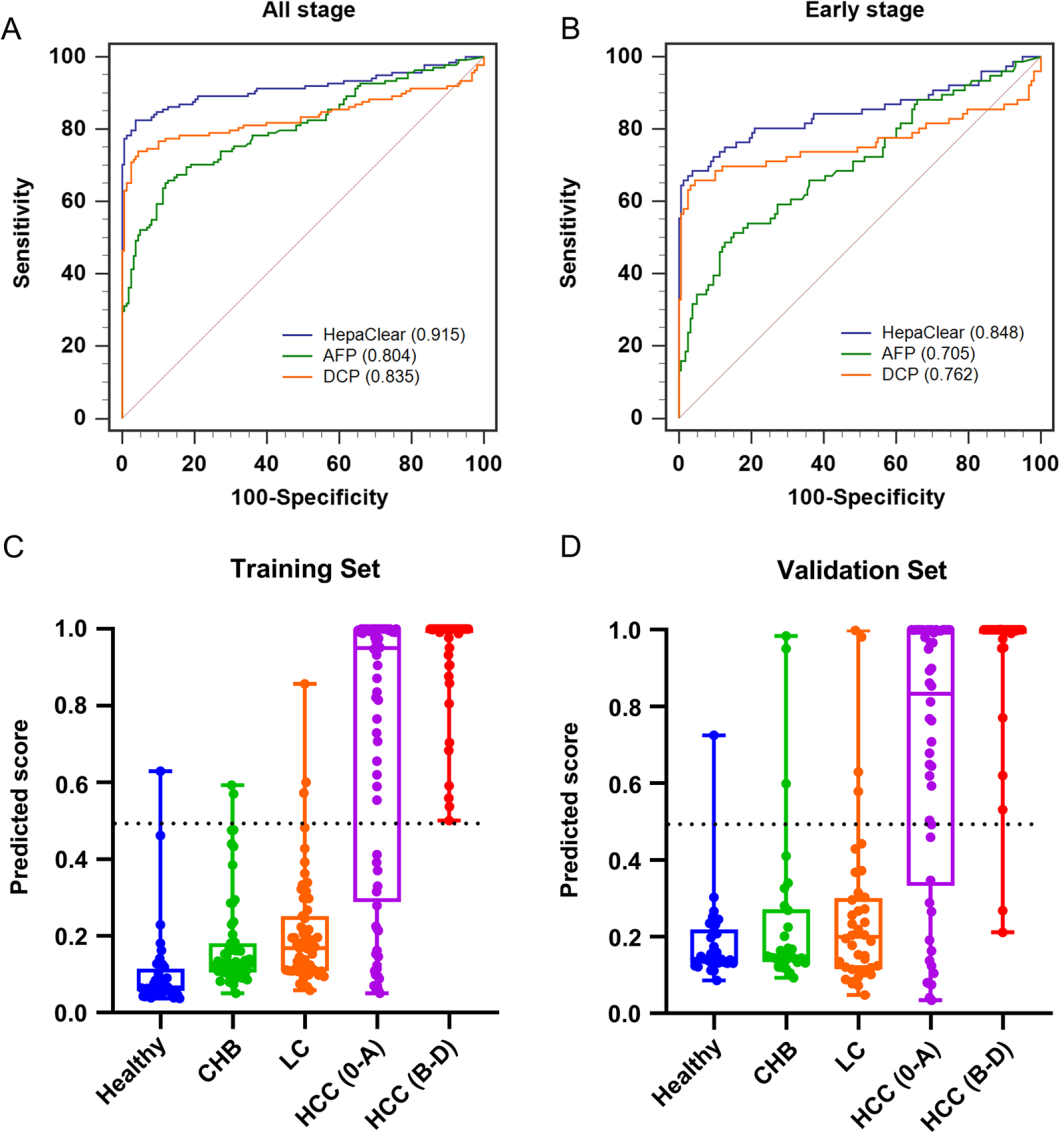
The performance of HepaClear in the training and validation sets. **A**, **B** ROC curves of the HepaClear panel compared with AFP and DCP alone in the training group among all-stage patients (**A**) and early-stage HCC patients (**B**). **C**, **D** Predicted score distributions among healthy controls, CHB, LC and HCC patients in the training group (**C**) and the validation group (**D**)

The Ct value of methylated CpG sites and log-transformed AFP/DCP levels from the training set were used to develop a logistic regression algorithm. As shown in Fig. 3A and Table 3, among 138 all-stage HCC patients in the training set, the HepaClear panel yielded an AUC value of 0.915 using a cutoff score of 0.481, with 82.6% (95% CI 75.2–88.5%) sensitivity at 96.2% (95% CI 91.9–98.6%) specificity. The novel HCC biomarker panel showed better performance than AFP at the clinical cutoff value of 20 ng/mL [31], which had a sensitivity of 52.2% (95% CI 43.5–60.7%) with 93.7% (95% CI 87.9–96.5%) specificity (Table 3). The DCP marker also showed lower performance than the HepaClear panel, with a sensitivity of 72.9% (95% CI 64.8–81.0%) and specificity of 92.0% (95% CI 86.9–95.5%) at a cutoff value of 40 mAU/mL (Table 3) [32]. Among 76 early-stage HCC patients in the training set, the HepaClear panel achieved an AUC value of 0.848 and sensitivity of 68.4% (95% CI 56.7–78.6%), which were higher than those of AFP (AUC: 0.705, 34.2% sensitivity, 95% CI 23.7–46.0%) and DCP (AUC: 0.762, 64.7% sensitivity, 95% CI 54.0–75.2%) (Fig. 3B, Table 3). The median value of the predicted score in the HCC groups was significantly higher than that in the normal groups (*p* < 0.01, Fig. 3C). Meanwhile, there was a gradually increasing trend of the predicted score from healthy, CHB, LC to HCC individuals, consistent with the development of HBV-related HCC.

**Table 3:** Performance of the HepaClear and comparison with AFP/DCP tests in the training set and the validation set

Marker(s) (cutoff)	Training set				Validation set			
	Specificity (95% CI) (%)	Sensitivity (95% CI) (%)			Specificity (95% CI) (%)	Sensitivity (95% CI) (%)		
		Stage 0-A (n = 76)	Stage B-D (n = 62)	All Stages (n = 138)		Stage 0-A (n = 50)	Stage B-D (n = 48)	All Stages (n = 98)
HepaClear (> 0.481)	96.2 (91.9–98.6)	68.4 (56.7–78.6)	100.0 (94.2–100.0)	82.6 (75.2–88.5)	92.0 (84.8–96.5)	72.0 (57.7–83.4)	95.8 (85.7–99.5)	84.7 (76.0–91.2)
AFP (> 20 ng/mL)	93.7 (87.9–96.5)	34.2 (23.7–46.0)	74.2 (61.5–84.5)	52.2 (43.5–60.7)	94.0 (87.4–97.8)	48.0 (33.7–62.6)	72.9 (58.2–84.7)	60.2 (49.8–70.0)
DCP (> 40 mAU/mL)	92.0 (86.9–95.5)	64.7 (54.0–75.2)	83.9 (72.3–92.0)	72.9 (64.8–81.0)	92.0 (84.8–96.5)	62.0 (46.2–74.6)	87.5 (74.8–95.3)	74.5 (63.6–81.9)

*AFP* a-fetoprotein, *CI* confidence interval, *DCP* des-gamma-carboxyprothrombin

To further validate the performance of the HCC diagnosis model based on the training set, we used the HepaClear assay on a validation set consisting of an independent cohort. At a cutoff value of 0.481, the HepaClear panel showed 84.7% (95% CI 76.0–91.2%) sensitivity and 92.0% (95% CI 84.8–96.5%) specificity. In contrast, the AFP single biomarker yielded a sensitivity of only 60.2% (95% CI 49.8–70%) and 94.0% (95% CI 87.4–97.8%) specificity using a 20 ng/mL cutoff (Table 3), while the DCP marker yielded a sensitivity of 74.5% (95% CI 63.6–81.9%) and 92.0% (95% CI 84.8–96.5%) specificity at a cutoff value of 40 mAU/mL. Among 50 early-stage HCC patients, the panel showed 72.0% (95% CI 57.7–83.4%) sensitivity, while AFP alone showed a sensitivity of only 48.0% (95% CI 33.7–62.6%), and DCP alone showed a sensitivity of 62.0% (95% CI 46.2–74.6%) (Table 3). Similar to the training set, the predicted scores in the HCC groups were much higher than those in the normal groups (*p* < 0.01, Fig. 3D) and showed a gradual increasing trend.

In addition, we evaluated the performance of the HepaClear panel within different subgroups of the validation set (Table 4). The sensitivity was 83.0% (95% CI 73.8–90.0%) in CHB patients and 85.6% (95% CI 76.7–91.7) in cirrhosis patients at 90% specificity. For AFP-negative HCC patients, 67.5% (95% CI 50.9–81.4%) obtained positive results using the HepaClear assay. Collectively, these results indicated the advantage of HepaClear over AFP and DCP in differentiating HCC and non-HCC individuals from the high-risk population.

**Table 4:** Sensitivity and specificity of HepaClear in different subgroups from the validation set

Subgroup	Sensitivity (95% CI) (%)	Specificity (95% CI) (%)
Male (n = 149)	85.4 (75.8–92.2)	91.0 (81.5–96.6)
CHB (n = 153)	83.0 (73.8–90.0)	89.2 (79.1–95.6)
Cirrhosis (n = 130)	85.6 (76.7–91.7)	90.0 (76.3–97.2)
AFP < 20 ng/mL (n = 134)	67.5 (50.9–81.4)	92.6 (85.3–97.0)

*AFP* a-fetoprotein, *CHB* chronic hepatitis B, *CI* confidence interval

## Discussion

We developed a cost-effective liquid biopsy HCC panel that demonstrated higher sensitivity and diagnostic accuracy than current biomarker-based tests, including AFP and US. The HepaClear panel showed higher sensitivity than AFP and DCP for early-stage and late-stage HCC. Additionally, the panel performed consistently in both the training set and validation set and yielded high accuracy in different subgroups, including viral etiology and cirrhosis, indicating a valuable opportunity for HCC detection during disease surveillance. In conclusion, our study shows that the HepaClear assay may have superior clinical performance than AFP and has potential for HCC surveillance of high-risk subjects.

Three methylated CpG sites (cg14263942, cg12701184 and cg14570307), for which the RefGenes are *CDKL2*, *USP44* and *ZNF783*, respectively, were finally chosen to build the HCC diagnosis model. *CDKL2* is a member of the cdc2-related serine/threonine kinase subfamily and negatively regulates the cell cycle [33]. *CDKL2* has been reported to be hypermethylated in HCC tissues, and the hypermethylation of *CDKL2* causes decreased CDKL2 mRNA expression [34]. Low expression of *CDKL2* is positively correlated with tumor cell proliferation and invasion [35]. *USP44* is a member of the deubiquitinating enzyme (DUB) family and is regarded as a key factor involved in DNA double-strand break repair as well as the regulation of mitotic spindle formation and centrosome positioning [36, 37]. It has been reported that USP44 hypermethylation promotes tumorigenesis and metastasis in multiple cancers [38–40]. In HCC, a lower level of USP44 expression in HCC samples predicted poor prognosis. ZNF783 belongs to the zinc finger protein (ZFP) family, which plays a significant role in HCC oncogenesis and progression as a transcription factor [41]. Although the mechanism and biological function of *ZNF783* hypermethylation in HCC tumorigenesis have not been reported, our data demonstrated that *ZNF783* could still be a potential DNA marker in HCC.

In the methylation biomarker discovery and validation phase, several criteria were established to minimize the potential false-positive and false-negative cases in the following plasma cfDNA assay. First, based on a high differential methylation level (≥ 0.3) between HCC and paired adjacent tissues, we selected candidate markers with an average methylation level of ≤ 0.1 to control for methylation signatures contributed from other liver diseases. Normal individuals with liver damage may display much higher levels of liver-derived cfDNA, which could reduce the specificity of liquid biopsy if candidate markers were not sufficiently hypomethylated in normal tissues. Second, we evaluated the performance of each candidate marker in discriminating HCC and control samples using AUC, sensitivity, specificity, and YI analysis. Identifying methylation markers in such criteria can avoid choosing inappropriate biomarkers, which show extremely high ∆β values in a small portion of paired tissues and low ∆β values in relatively more tissues. Third, in addition to adjacent normal tissues, we also used buffy coat tissue (Additional file 1: Fig. S3) as a control sample during biomarker tissue validation. Since leukocyte-derived DNA constitutes most of the plasma circulating DNA pool, even trace amounts of methylated target sites may cause false-positive results.

Although the HepaClear assay showed 82–85% sensitivity for all-stage HCC at ≥ 92% specificity, ~ 30% of early-stage HCC cases were missed in both the training and validation cohorts. One possible reason can be attributed to the bottleneck of the ctDNA methylation detection method. For some early-stage HCC patients, ctDNA comprises a very small fraction (< 1%) of cfDNA [42] and the actual fraction of methylated DNA fragments would easily fall below the limit of detection of qMSP if cfDNA loss during preanalytical sample processing is considered. On the other hand, HepaClear yielded 14 false-positive cases among the two cohorts. Among these cases, 6 had abnormally elevated AFP or DCP levels, and 10 showed elevated methylation signals from at least one marker gene. In addition to technical errors, these abnormal marker signals may arise as a driver or a consequence of physiological disorder. We tracked the fourteen individuals with false-positive results for six months, seven cases were lost and no new clinical information was obtained. Of the remaining seven individuals who completed follow-up, one developed HCC and the others were consistent with the original case information (hepatitis B or cirrhosis).

Over recent years, many HCC diagnosis models based on cfDNA methylation and protein biomarkers have been reported [43]. Chalasani et al. reported a multitarget HCC panel integrating 4 DNA methylation markers and 2 protein markers, with a sensitivity of 80% for any-stage HCC and 71% for early-stage HCC at 90% specificity. Our HepaClear panel yielded similar sensitivity and specificity as the multitarget HCC panel, and the model performance was validated in an independent cohort. Compared to the study from Chalasani et al*.*, in which hepatitis C virus (HCV)- or nonalcoholic fatty liver disease (NAFLD)-related cases were enrolled, our study indicates that the HepaClear panel is more applicable to Chinese high-risk populations because over 80% of HCC patients in China were diagnosed as HBV-positive [44]. Luo et al*.* [45] presented an HCC screening model based on 2321 methylation markers, and the screening model achieved a sensitivity of 84% and specificity of 96% in the validation set. However, cancer screening assays based on next-generation sequencing (NGS) are time-consuming, while the HepaClear assay has the advantages of low financial cost (< 10 dollars per test) and low time cost (1 day from sample processing to data analysis). Furthermore, the DNA methylation detection technology used in our study was the 850K Methylation BeadChip array, which provides more comprehensive coverage of genome-wide methylation sites (> 850,000 methylation sites) than the traditional 450K Methylation BeadChip array. cg12701184, which is included in HepaClear, is a novel methylated site detected by the 850K Methylation BeadChip array. Therefore, as a time-efficient assay with novel methylated CpG sites showing high sensitivity and specificity, HepaClear could have comparable clinical applicability in the Chinese high-risk population. Our results are consistent with a previous study that showed the superiority of DCP to AFP in HCC surveillance and strengthen the viewpoint that DCP has higher sensitivity of detecting HCC with an HBV-positive background.

Despite the encouraging results, there are still limitations to our study. First, the sensitivity of HepaClear in the early stage needs to be improved by optimizing sample processing and adding new biomarkers. Second, the CHB/LC group and healthy group had shorter median ages than the HCC group in both the training and validation cohorts, which may have influenced the biomarker levels. Third, the novel methylated site detected by the 850K Methylation BeadChip array in our study lacks verification from public data. Few data from the 850K Methylation BeadChip array are currently accessible in online databases, such as TCGA or GEO. Therefore, additional studies, including algorithm optimization and prospective, multicenter clinical validation, will further demonstrate the robustness, efficiency, and cost-effectiveness of the HepaClear assay as an HCC screening tool.

## Conclusions

In summary, we have developed HepaClear, a novel noninvasive HCC screening assay that integrates DNA methylation markers and protein markers. HepaClear can be effectively implemented on HCC high-risk (CHB/LC) individuals with favorable performance and low cost. Additional studies should be performed to demonstrate the better clinical sensitivity and utility of the HepaClear assay than US plus AFP in future clinical applications.

## Methods

### Participants and sample collection

In this retrospective study, 644 participants were enrolled from September 2019 to March 2022, including 100 healthy individuals, 119 CHB patients, 129 LC patients and 296 HCC patients. Participants were recruited from three institutions: the Department of Hepatic Surgery, Tianjin First Central Hospital (*n* = 246), the Department of Hepatic Surgery, Peking Union Medical College Hospital (*n* = 248), and Department of Infection, Central Hospital of Shengli Oilfield (*n* = 150).

Tissues used in biomarker discovery and validation were obtained at the time of surgical operation from HCC patients without any previous treatment, including 80 paired HCC tumor and adjacent nontumor liver tissue samples. All tissue samples within this study were stored at − 80 °C before use, and tumor tissues underwent histopathological evaluation before DNA extraction. Sixty paired tissues were selected for 850K Methylation BeadChip array. Forty of 60 paired tissues, with an additional 20 pairs, were further used for the TaqMan qMSP assay to validate the biomarkers.

Overall, 644 blood samples from the abovementioned participants were obtained, and those from HCC patients were collected after definite diagnosis and before treatment. Peripheral blood (5 mL, collected in EDTA tubes) was centrifuged twice at 1500× *g* for 15 min within 4 h of collection to isolate plasma, which was then stored at − 80 °C until experimental analysis. In most cases, 2 mL of plasma was used for cfDNA extraction, and 0.5 mL plasma was used to measure the concentrations of AFP and DCP.

### Biomarker discovery and validation

**Fig. 4:**
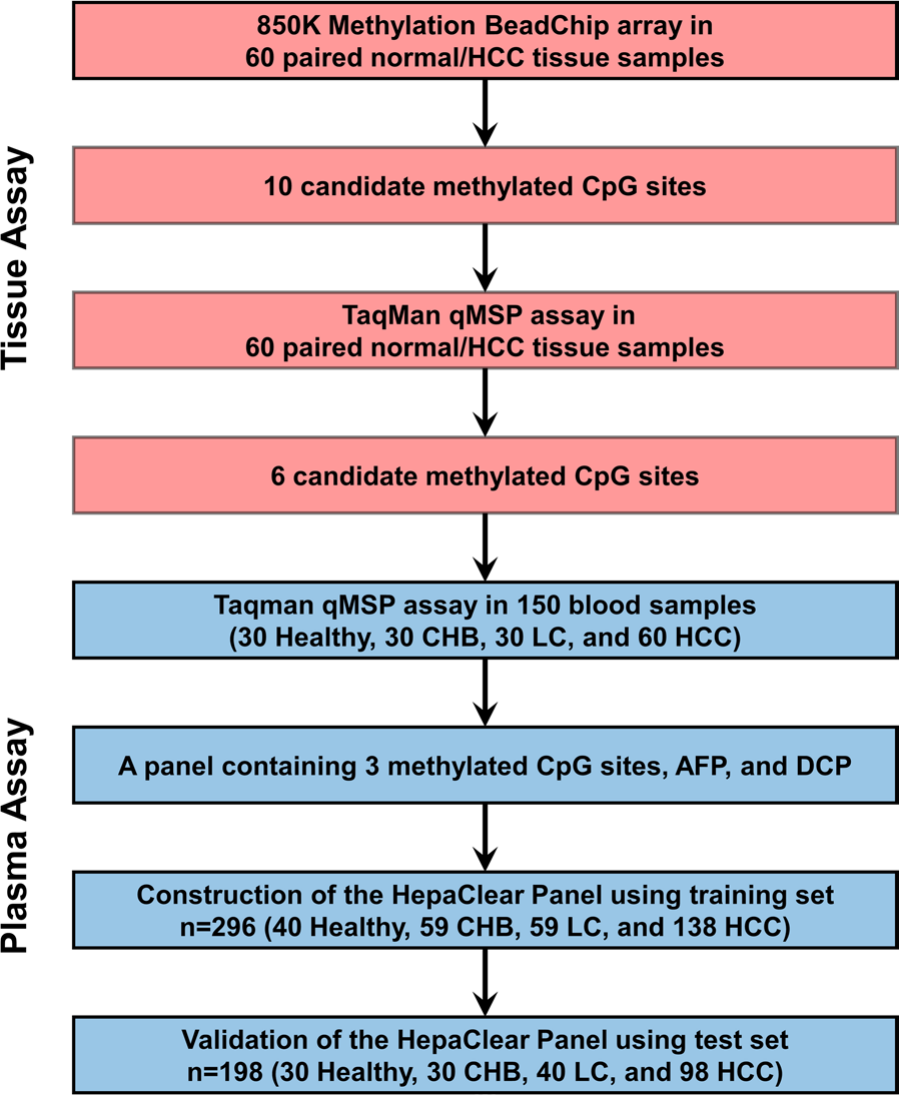
Flow diagram of the study

This study was performed in four sequential procedures, according to the flow diagram shown in Fig. 4. First, we used a 850K Methylation BeadChip array (Illumina, San Diego, CA) to detect altered methylation regions in tissue DNA and select candidate hypermethylated CpG sites in HCC tissue DNA. Methylation 450K BeadChip array datasets collected from The Cancer Genome Atlas (TCGA) and the Gene Expression Omnibus (GEO) database were used for comparison with the results of our study. Second, quantitative methylation-specific PCR (qMSP) was used for tissue validation, and HCC tissue-specific differential methylation sites were further confirmed. Third, selected methylated CpG sites, with HCC protein markers, were tested on 150 plasma samples for the pilot study. Area under the ROC curve (AUC) values and Youden index (YI) at the corresponding biomarker combinations were evaluated. Finally, the selected methylated CpG sites were tested by TaqMan qMSP assay on blood samples in the two groups. A blood-based panel of DNA methylated CpG sites and protein markers (AFP, DCP) was constructed based on the data in the training set and further evaluated based on the data in the validation set.

### Tissue/cell/plasma DNA extraction and bisulfite conversion

For tissue and cell samples, genomic DNA was extracted using a QIAamp DNA Mini Kit (Qiagen, Hilden, Germany) according to the manufacturer’s instructions. Circulating cfDNA from ~ 2 mL plasma samples was extracted using the Magnetic Serum/Plasma Circulating DNA Maxi Kit (TianGen Biotech, Beijing). Tissue and cell DNA was quantified with a NanoDrop 2000 Spectrophotometer (ThermoFisher Scientific, Waltham, MA), and plasma cfDNA was quantified with a Qubit 4.0 fluorometer (ThermoFisher Scientific, Waltham, MA). For bisulfite conversion, 500 ng of tissue DNA or 40 μL of plasma cfDNA was converted according to the manufacturer’s instructions using an EZ DNA Methylation-Gold Kit (Zymo Research, Irvine, CA).

### Methylation BeadChip array

Converted DNA was used for hybridization on an Illumina Infinium 850K Methylation BeadChip array (Illumina, San Diego, CA), and an Illumina HD methylation assay kit (Shanghai Biotechnology Corporation) was used to assay DNA methylation levels. After quality filtering and data normalization, differentially methylated CpGs between HCC and normal liver tissues were screened using the R package IMA. We used the mean difference in β-value (Δβ) between the two abovementioned groups to evaluate the methylation level for each CpG site. CpG sites that showed |∆β|> 0.1 and *p* value < 0.05 were defined as significantly differentially methylated sites.

### TaqMan qMSP for biological tissue validation

For validation of candidate methylated sites, tissue DNA samples were used for qMSP based on a TaqMan probe. The total volume of the qMSP reaction was 20 μL, containing 2 μL bisulfite-converted gDNA and 1 unit TaKaRa Taq™. DNA samples were assayed on an ABI7500 (Applied Biosystems, Foster City, CA) under the following conditions: 94 °C for 3 min, 45 cycles of 94 °C for 30 s and 60 °C for 35 s. Positive control (PC) and negative control (NC) were included in each plate. PC was composed of HepG2 cell DNA, while NC was composed of HIE-2 cell DNA. PCR results were normalized to *β-2-microglobulin* (*B2M*) amplified from the same sample, and the ΔCt of each sample was calculated for statistical analysis. Primers and probes for qMSP are summarized in Additional file 1: Table S8.

### Plasma biomarker detection

For the methylation marker assay, 10 μL bisulfite-converted cfDNA was amplified in a volume of 30 μL containing 2 units Platinum™ Taq DNA Polymerase, and qPCR was performed with an ABI 7500 system (Applied Biosystems, Foster City, CA) as above. qMSP was performed in triplex assays (2 methylation markers plus internal reference gene *B2M*) for biomarker verification and in quadruplex assays (3 methylation markers plus *B2M*) for model training and validation. PC (Huh7 cell DNA) and NC (HIE-2 cell DNA) were also included, in qMSP. The experiments with plasma samples were performed in a blinded fashion.

### Limit of detection (LOD) of quadruplex methylation biomarkers assay

LOD assay of three methylation biomarkers were performed using NC and PC DNA. NC was composed of HIE-2 cell DNA, while PC was composed of Huh7 cell DNA spiked in HIE-2 cell DNA at ratios of 5%, 1%, 0.5% or 0.25%. All the three methylation biomarkers were found methylated in Huh7 cell DNA, and unmethylated in HIE-2 cell DNA using sanger sequencing. Both PC and NC DNA were diluted to 2 ng/μL before use. For LOD assays, NC and different PCs were converted using an EZ DNA Methylation-Gold Kit (Zymo Research, Irvine, CA), and qMSP with a 30 μL reaction system containing 5 μL DNA template was performed with an ABI 7500 system (Applied Biosystems, Foster City, CA). Each bisulfite-treated DNA sample were assayed in twenty replicate wells.

### Statistical analysis

Differences between groups were analyzed using the two-tailed Student’s t test, the Kruskal‒Wallis test, or the chi-square test, where appropriate. A *p* value < 0.05 was regarded as statistically significant. To evaluate the clinical performance of each biomarker, receiver operating characteristic (ROC) curves were constructed, and the cutoff for each candidate marker was determined based on the YI. In the plasma pilot study, DNA methylation marker levels, protein marker levels and clinical information were used for HCC diagnosis model construction and validation. The Ct value of methylated CpG sites and the log-transformed protein values were collected before data input. All Ct values of undetected methylation markers from qMSP were rounded up to 45, and log-transformed AFP and DCP values (calculated as < 0) were rounded up to 0. Logistic regression was used for constructing models of marker combinations, and the logistic regression algorithm developed from HepaClear panal was as follows: score = 30.9062 − 0.3385 × cg14263942 − 0.2114 × cg12701184 − 0.2202 × cg14570307 + 0.3362 × log_2_AFP + 0.2656 × log_2_DCP. All statistical tests were performed with the Statistical Program for Social Sciences (SPSS 20.0 for Windows, USA) or GraphPad Prism 7 Software (GraphPad, USA). GraphPad Prism 7 and MedCalc Statistical Software version 20.0.4 (MedCalc Software Ltd, Ostend, Belgium) were used for the creation and analysis of graphs.

## Supplementary Information

**Additional file 1. Figure S1.** GO and KEGG pathway enrichment analysis of differentially methylated CpG sites. **Figure S2. **Performance of 32 candidate CpG sites identified from methylome profiling data. **Figure S3.** Methylation levels of 10 candidate CpG sites in two HCC cell lines and leukocytes using qMSP. **Figure S4.** Methylation/Protein levels and diagnostic performance of eight candidate markers in plasma pilot test set. **Figure S5.** Amplification curves of cg14263942, cg12701184 and cg14570307 within quadruplex assays. **Table S1.** Demographic and clinical characteristics of HCC patients for biomarker screening and tissue validation. **Table S2.** Top-1000 hypermethylation markers screened from genome-wide methylation profiling. **Table S3.** List of 132 hypermethylation markers with *p* < 0.05, Δβ > 0.3 and β_normal_ < 0.1. **Table S4.** List of 32 markers further screened from Table S3, with AUC > 0.85 and Youden Index (YI) ≥ 0.8. **Table S5.** Characteristics of study participants in plasma pilot study. **Table S6.** Performance of different biomarker combinations in 150 plasma samples. **Table S7.** Limit of detection (LOD) of three methylation markers in HepaClear panel. **Table S8.** List of primers and probes for Taqman qMSP.

## Data Availability

The datasets used and/or analyzed during the current study are available from the corresponding author on reasonable request.

## Abbreviations

HCC: Hepatocellular carcinoma
qMSP: Quantitative methylation-specific PCR
AFP: α-Fetoprotein
DCP: Des-gamma-carboxyprothrombin
APASL: Asian Pacific Association for the Study of the Liver
HBV: Hepatitis B virus
HCV: Hepatitis C virus
LC: Liver cirrhosis
US: Ultrasonography
BCLC: Barcelona Clinic Liver Cancer
CHB: Chronic hepatitis B
cfDNA: Cell-free DNA
ctDNA: Circulating tumor DNA
TCGA: The Cancer Genome Atlas
GEO: Gene Expression Omnibus
ROC: Receiver operating characteristic
AUC: Area under ROC curve
YI: Youden index
GO: Gene ontology
KEGG: Kyoto Encyclopedia of Genes and Genomes
B2M: β-2-Microglobulin
CDKL2: Cyclin-dependent kinase-like 2
USP44: Ubiquitin specific peptidase 44
ZNF783: Zinc finger family member 783
NAFLD: Non-alcoholic fatty liver disease
NGS: Next generation sequencing
PC: Principal component
