# Supplementary material for: HepaClear, a blood-based panel combining novel methylated CpG sites and protein markers, for the detection of early-stage hepatocellular carcinoma
Source: Clin Epigenetics. 2023 Jun 12;15:99. doi: 10.1186/s13148-023-01508-7 (PMC10258978; doi:10.1186/s13148-023-01508-7)
Supplement: Supplementary file 1 — Additional file 1. Figure S1. GO and KEGG pathway enrichment analysis of differentially methylated CpG sites. Figure S2. Performance of 32 candidate CpG sites identified from methylome profiling data. Figure S3. Methylation levels of 10 candidate CpG sites in two HCC cell lines and leukocytes using qMSP. Figure S4. Methylation/Protein levels and diagnostic performance of eight candidate markers in plasma pilot test set. Figure S5. Amplification curves of cg14263942, cg12701184 and cg14570307 within quadruplex assays. Table S1. Demographic and clinical characteristics of HCC patients for biomarker screening and tissue validation. Table S2. Top-1000 hypermethylation markers screened from genome-wide methylation profiling. Table S3. List of 132 hypermethylation markers with p < 0.05, Δβ > 0.3 and βnormal < 0.1. Table S4. List of 32 markers further screened from Table S3, with AUC > 0.85 and Youden Index (YI) ≥ 0.8. Table S5. Characteristics of study participants in plasma pilot study. Table S6. Performance of different biomarker combinations in 150 plasma samples. Table S7. Limit of detection (LOD) of three methylation markers in HepaClear panel. Table S8. List of primers and probes for Taqman qMSP. [file 13148_2023_1508_MOESM1_ESM.docx]

**Addition file 1:**

**Supplementary method**

**Participants’ enrollment**

For participants’ enrollment, eligible subjects 18-75 years old were invited to participate in this study. Subjects then received clinical and biochemical evaluations and liver imaging, including serum AFP concentration, hepatitis B surface antigen (HBsAg) level and US. Individuals who either had serum AFP level ≥ 400 ng/mL or had a lesion (≥ 1 cm in size) detected by US were referred to further diagnosis, and HCC was ascertained by either dynamic computed tomography (CT), multiphase contrast-enhanced magnetic resonance imaging (MRI), or biopsy. All 4 BCLC stages were included, while over half of HCC patients were classified as BCLC stage 0 or A. Non-HCC patients with cirrhosis or CHB were recruited in control group if the result of AFP and US testing showed no evidence of HCC during blood sampling. The healthy individuals in this study were derived from those who did physical examination and had neither HBsAg nor cirrhosis positive results. Subjects who had other malignant tumors including intrahepatic cholangiocarcinoma (ICC) or had received cancer treatment (surgery, chemotherapy, radiotherapy) within less than 3 years were excluded from this study.

**Cell culture**

Two HCC cell lines, HepG2 and Huh7, were kindly provided by Stem Cell Bank, Chinese Academy of Sciences (CAS). The intestinal mucosa-derived cell line, HIE-2, was kindly provided by Cell Resource Center, IBMS, CAMS/PUMC. HepG2 and Huh7 cells were cultured in DMEM (Invitrogen, USA) supplemented with 10% FBS (Gibco, USA). HIE-2 cell was cultured in RPMI 1640 Medium (Invitrogen, USA) supplemented with 10% FBS (Gibco, USA).

**Methylation array data analysis**

For further validation of candidate hypermethylation markers, two external datasets were used. First, 450K methylation array dataset LIHC, including 377 HCC and 50 normal tissue samples, was obtained from TCGA public database using the XENA browser^[1].^ Second, a methylation array dataset, GSE56588 (244 HCC and 19 non-HCC), was collected from the Gene Expression Omnibus (GEO) database^[2]^. In each dataset, probes significantly hypermethylated in HCC group (Δβ > 0.1 and P value < 0.05) were selected and ranked by Δβ. Then, we divided the 850K methylation array data from 60 pairs of tissue samples into two subgroups: Group A contains 450K methylation loci, while group B contains loci in 850K dataset but not in 450K dataset. We selected Top 1000 probes from TCGA-LIHC, GSE56588 and Group A, and then merged them to select overlapping markers for further analysis.

**Determination of plasma AFP and DCP concentrations**

Plasma AFP and DCP levels were measured using commercial kits in the Abbott ARCHITECT i2000SR Chemical luminescence immunity analyzer (CLIA) according to the manufacturer’s instructions (Abbott Laboratories; Chicago, IL, USA).

1. Goldman MJ, Craft B, Hastie M, Repecka K, McDade F, Kamath A, et al. Visualizing and interpreting cancer genomics data via the Xena platform. Nat Biotechnol. 2020;38(6):675-8.

2. Villanueva A, Portela A, Sayols S, Battiston C, Hoshida Y, Mendez-Gonzalez J, et al. DNA methylation-based prognosis and epidrivers in hepatocellular carcinoma. Hepatology. 2015;61(6):1945-56.


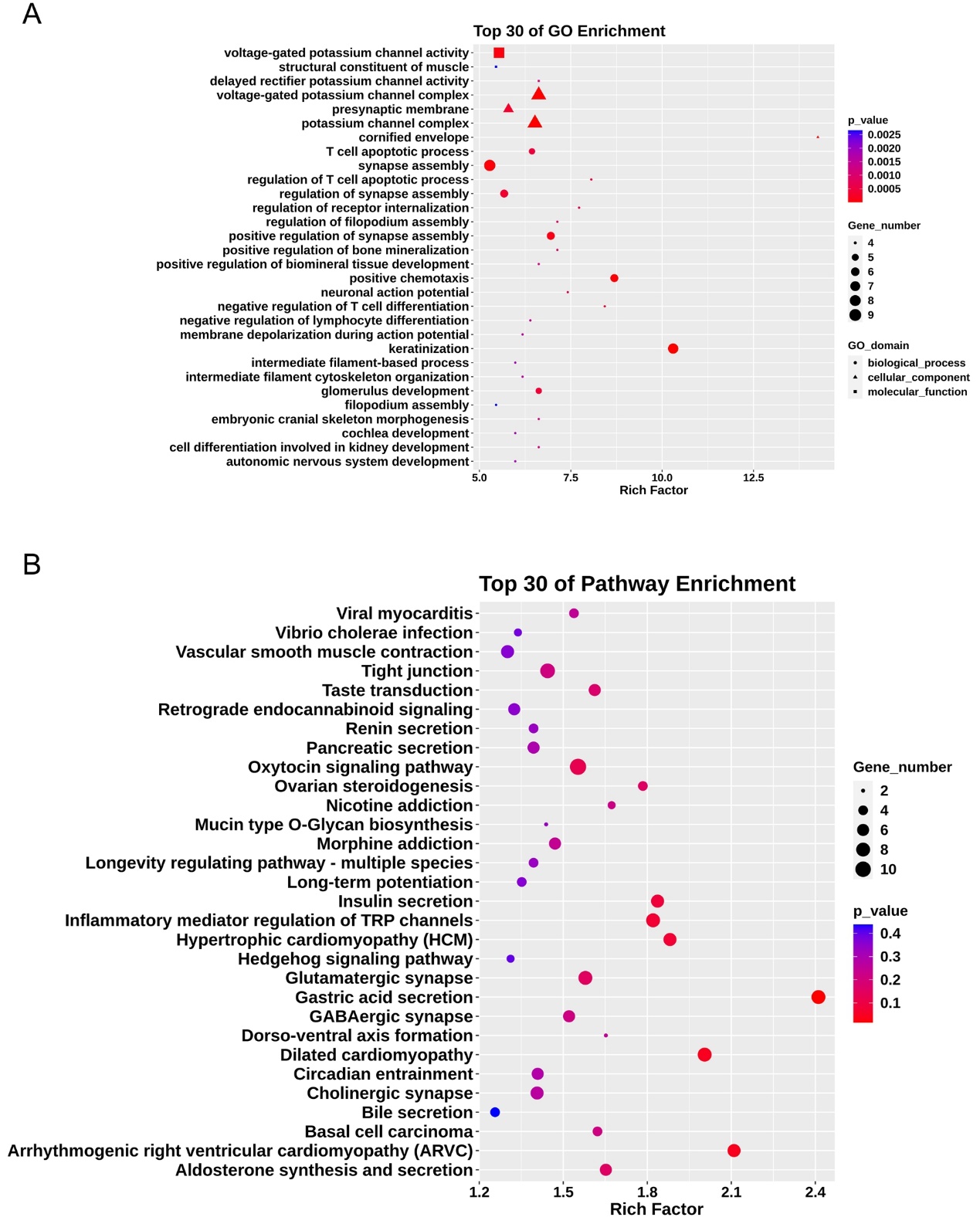


**Figure S1. GO and KEGG pathway enrichment analysis of differentially methylated CpG sites.** (A) The top 30 terms in Gene Ontology (GO) analysis. (B) The top 30 pathways in Kyoto Encyclopedia of Genes and Genomes (KEGG) analysis. ‘Rich factor’ is the percentage of CpG sites in the given GO term/KEGG pathway. The color and size of each bubble represent enrichment significance and the number of methylated CpG sites enriched in a GO term or pathway.


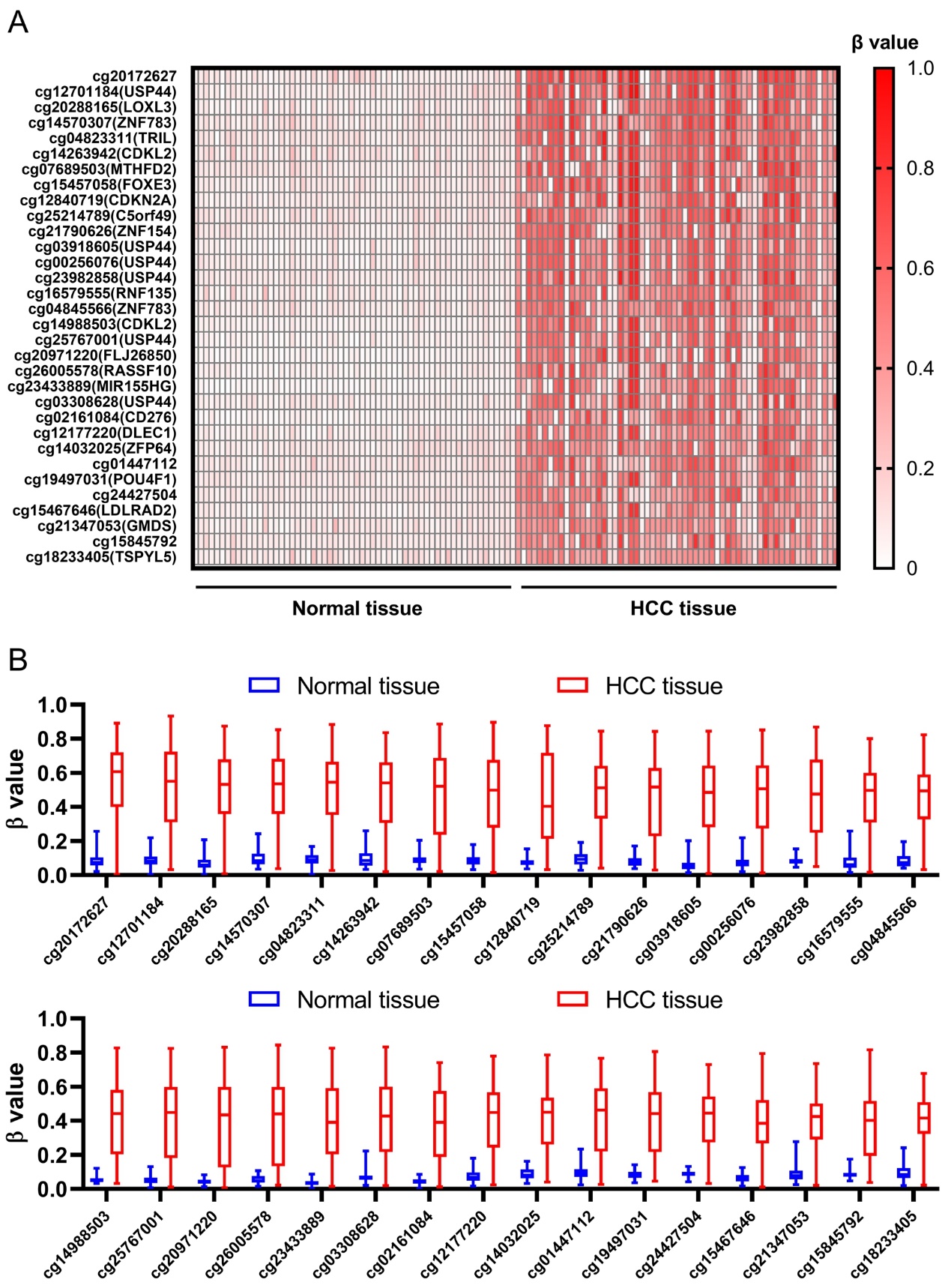


**Figure S2. Performance of 32 candidate CpG sites identified from methylome profiling data.** (A) A heat map showing methylation level of 32 candidate CpG sites in 60 pairs of HCC and normal tissue samples. Each column represents a tissue sample, and each row represents a methylated CpG site. (B) The beta value of 32 CpG sites in 60 pairs of HCC and normal tissue samples.


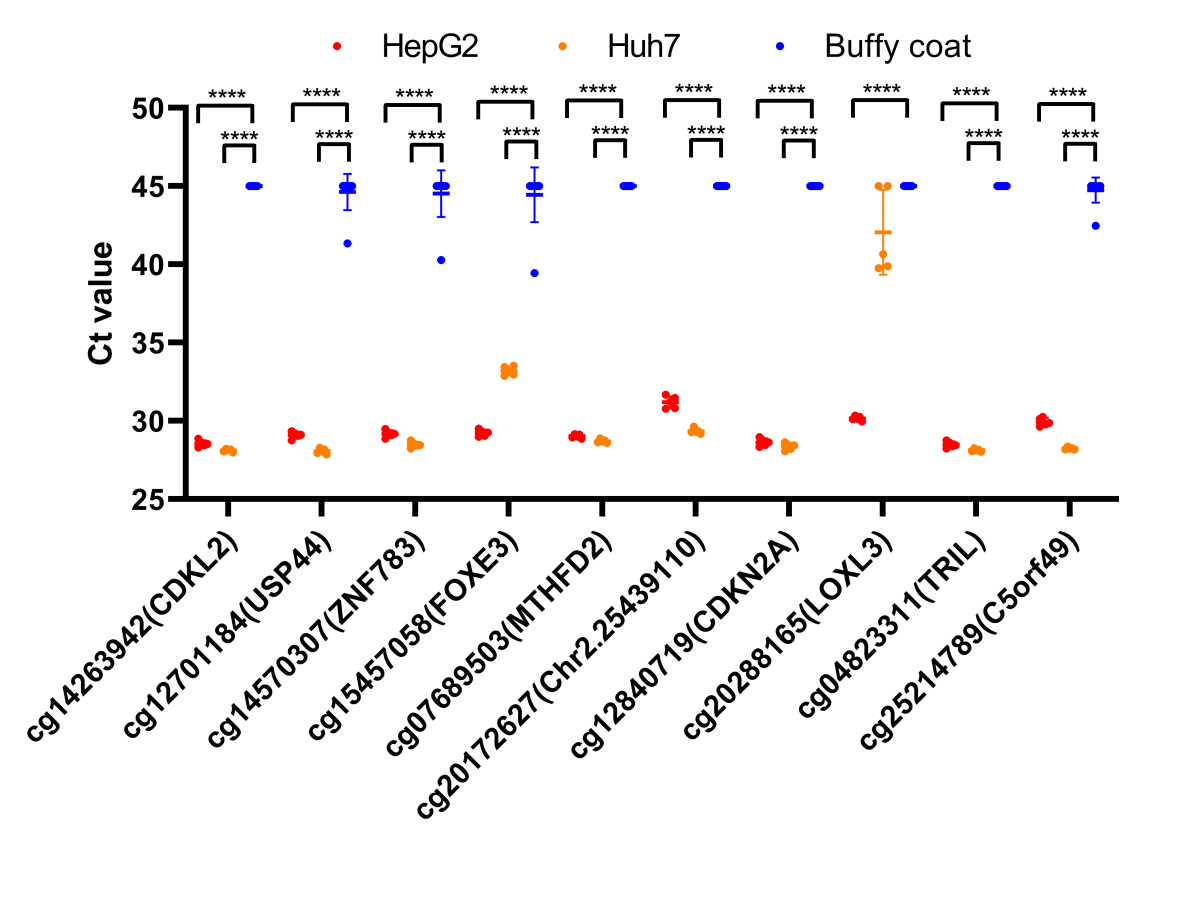


**Figure S3. Methylation levels of 10 candidate CpG sites in two HCC cell lines and leukocytes using qMSP.** For each candidate CpG sites, DNA extracted from 5 tubes of HepG2 cells, 5 tubes of Huh7 cells and 10 tubes of buffy coats were separately assay for evaluating methylation levels. Ct values of undetected methylation markers from qMSP were rounded up to 45. *B2M* were used as internal control, and Ct*_B2M_* ranged from 27.5 to 29.5 for each sample. *****p* < 0.0001.


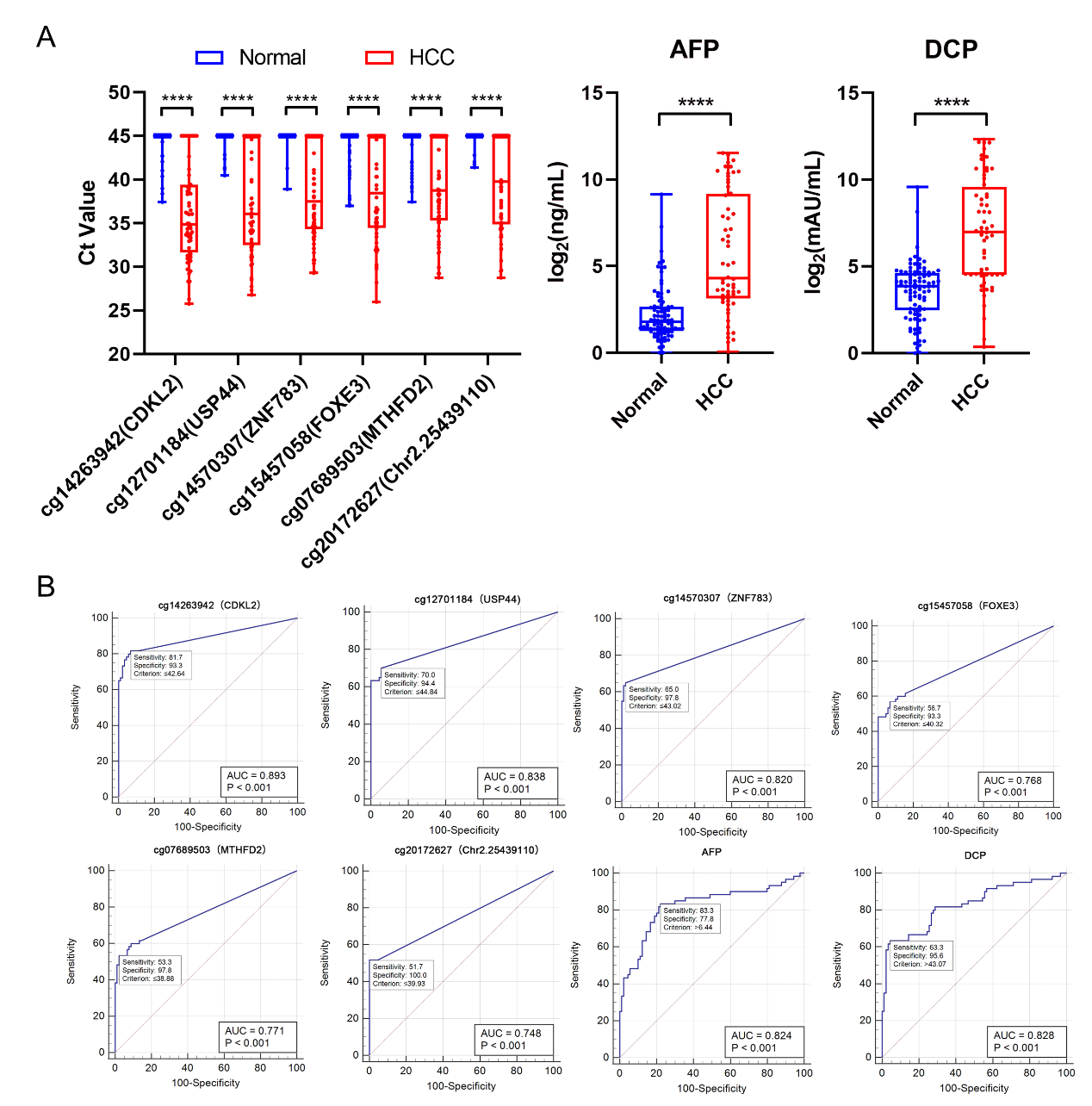


**Figure S4. Methylation/Protein levels and diagnostic performance of eight candidate markers in plasma pilot test set.** (A-C) Quantification of methylated CpG sites and marker proteins in different assays. Methylation levels of cfDNA (A) were quantified by qMSP while AFP (B) and DCP (C) levels were quantified by chemical luminescence immunity assay. The x-axis represents candidate sites/proteins, while the y-axis represents Ct value for candidate sites and log_2_(AFP/DCP) for protein markers. AFP was expressed as ng/mL, and DCP was expressed as mAU/mL. *****p* < 0.0001. (D) The ROC curves of each methylation marker and protein marker for separating HCC patients (n = 60) and normal controls (n = 90).


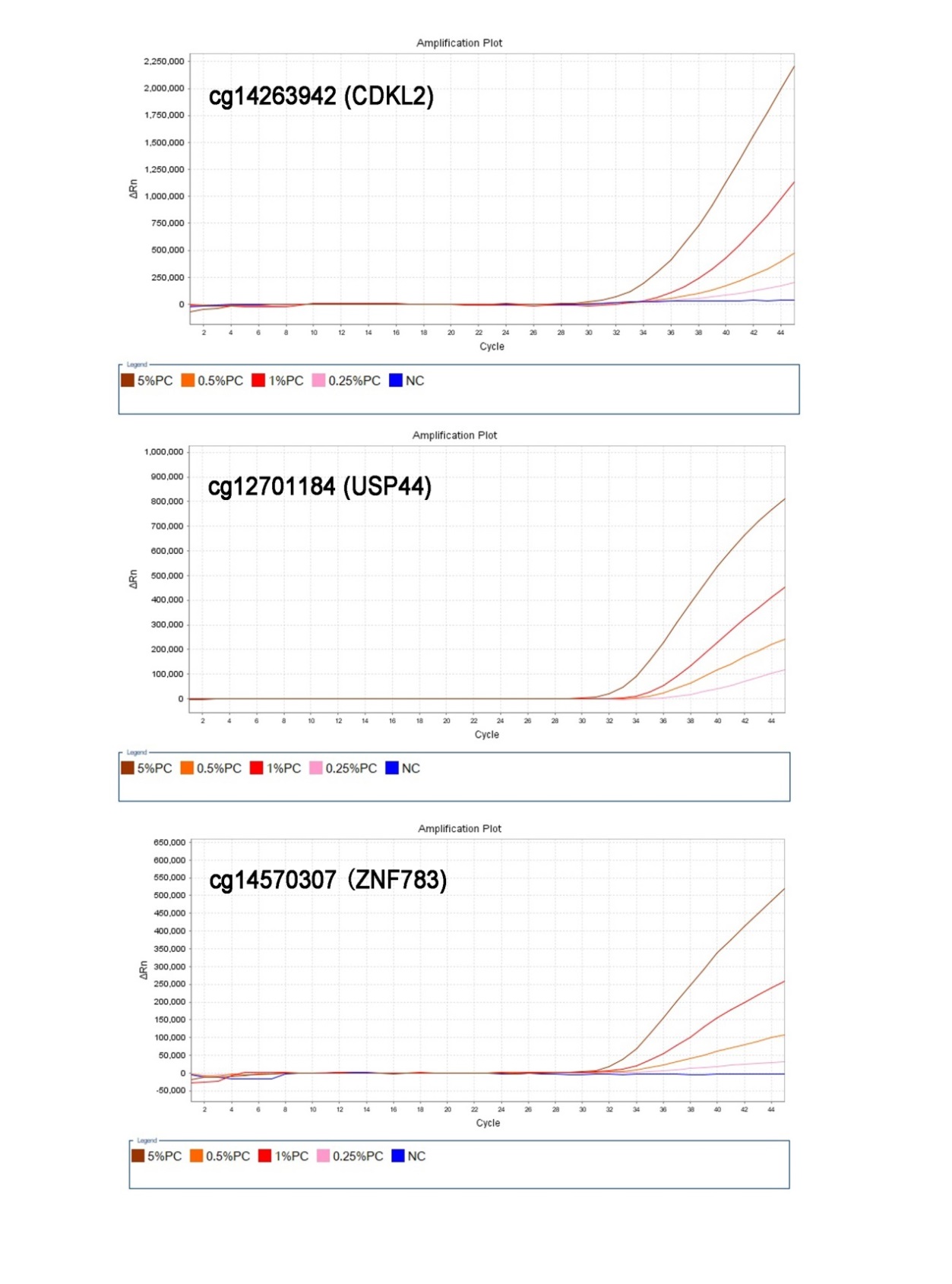


**Figure S5. Amplification curves of cg14263942, cg12701184 and cg14570307 within quadruplex assays.** Positive controls (PC) were composed of HIE-2 cell DNA and different concentrations of Huh7 cell DNA, while negative controls (NC) were composed of HIE-2 cell DNA.

**Table S1**. **Demographic and clinical characteristics of HCC patients for biomarker screening and tissue validation.**

| Characteristic and Statistic | | Overall (n = 80) | Individuals for marker screening (n = 60) | Additional individuals for marker validation (n = 20) |
| --- | --- | --- | --- | --- |
| Age (year), median (IQR) | | 57 (51-65) | 56 (49-63) | 62 (54-68) |
| Gender | |  |  |  |
|  | male (%) | 59 (73.8) | 47 (78.3) | 12 (60) |
|  | female (%) | 21 (26.2) | 13 (21.7) | 8 (40) |
| Etiology | |  |  |  |
|  | HBV (%) | 55 (68.8) | 41 (68.3) | 14 (70) |
|  | Alcohol (%) | 13 (16.3) | 10 (16.7) | 3 (15) |
|  | NAFLD (%) | 9 (11.2) | 7 (11.7) | 2 (10) |
|  | Other (%) | 3 (3.7) | 2 (3.3) | 1 (5) |
| Cirrhosis (%) | | 66 (82.5) | 46 (76.7) | 20 (100) |
| AFP (ng/mL) | |  |  |  |
|  | Median (IQR) | 9.59 (4.15-90.47) | 12.66 (6.58-144.6) | 10.72 (4.32-99.02) |
|  | < 20 (%) | 47 (58.8) | 35 (58.3) | 12 (60) |
|  | 20 - 400 (%) | 27 (33.7) | 22 (36.7) | 5 (25) |
|  | > 400 (%) | 6 (7.5) | 3 (5) | 3 (15) |
| BCLC stage | |  |  |  |
|  | 0 (%) | 4 (5) | 4 (6.7) | 0 (0) |
|  | A (%) | 53 (66.3) | 40 (66.7) | 13 (65) |
|  | B (%) | 15 (18.7) | 11 (18.3) | 4 (20) |
|  | C (%) | 8 (10) | 5 (8.3) | 3 (15) |
| Tumor size (cm) | |  |  |  |
|  | Median (range) | 4.8 (1.5-13.0) | 4.8 (1.5-11.2) | 5.5 (2.7-13.0) |
|  | < 3.0 (%) | 12 (15) | 10 (16.7) | 2 (10) |
|  | 3.0 - 5.0 (%) | 27 (33.8) | 22 (36.7) | 5 (25) |
|  | > 5.0 (%) | 31 (38.7) | 23 (38.3) | 8 (40) |
|  | Missing (%) | 10 (12.5) | 5 (8.3) | 5 (25) |
| Degree of tumour differentiation | |  |  |  |
|  | Well (%) | 14 (17.5) | 11 (18.3) | 3 (15) |
|  | Moderate (%) | 41 (51.3) | 31 (51.7) | 10 (50) |
|  | Poor (%) | 15 (18.7) | 13 (21.7) | 2 (10) |
|  | Missing (%) | 10 (12.5) | 5 (8.3) | 5 (25) |

**Table S2. Top-1000 hypermethylation markers screened from genome-wide methylation profiling.**

| Target ID | 850K  Beta.Diff. | Methyl450  Loci | TCGA  Beta.Diff. | GSE56588  Beta.Diff. |
| --- | --- | --- | --- | --- |
| cg22524657 | 0.515 | TRUE | 0.527 | 0.544 |
| cg20172627 | 0.463 | TRUE | 0.539 | 0.527 |
| cg22538054 | 0.462 | TRUE | 0.436 | 0.502 |
| cg01753936 | 0.462 | NA | NA | NA |
| cg19951303 | 0.462 | NA | NA | NA |
| cg01566592 | 0.444 | NA | NA | 0.581 |
| cg12539796 | 0.442 | TRUE | 0.420 | 0.486 |
| cg25851803 | 0.432 | NA | NA | 0.315 |
| cg22399133 | 0.430 | TRUE | 0.576 | 0.522 |
| cg25577023 | 0.429 | TRUE | 0.394 | 0.458 |
| cg00487232 | 0.426 | NA | NA | 0.422 |
| cg12701184 | 0.423 | NA | NA | NA |
| cg22035501 | 0.422 | TRUE | 0.452 | 0.514 |
| cg23947326 | 0.421 | NA | NA | 0.525 |
| cg13671044 | 0.418 | NA | NA | NA |
| cg11613015 | 0.417 | TRUE | 0.376 | 0.466 |
| cg24563094 | 0.416 | TRUE | 0.451 | 0.464 |
| cg01123334 | 0.416 | NA | NA | NA |
| cg02598441 | 0.413 | TRUE | 0.344 | 0.419 |
| cg26864230 | 0.412 | TRUE | 0.385 | 0.377 |
| cg06611810 | 0.410 | TRUE | 0.414 | 0.468 |
| cg09897199 | 0.410 | NA | NA | NA |
| cg15460872 | 0.410 | TRUE | 0.427 | 0.472 |
| cg25744484 | 0.410 | NA | NA | NA |
| cg13879483 | 0.409 | TRUE | 0.567 | 0.516 |
| cg20288165 | 0.405 | NA | NA | NA |
| cg03667968 | 0.404 | TRUE | 0.368 | 0.484 |
| cg10344081 | 0.403 | TRUE | 0.383 | 0.416 |
| cg26173997 | 0.403 | TRUE | 0.359 | 0.400 |
| cg05327835 | 0.403 | TRUE | 0.429 | 0.519 |
| cg21195185 | 0.402 | TRUE | 0.301 | 0.328 |
| cg10431993 | 0.402 | NA | NA | NA |
| cg14570307 | 0.402 | TRUE | 0.325 | 0.381 |
| cg14186292 | 0.401 | NA | NA | NA |
| cg18771357 | 0.401 | TRUE | 0.390 | 0.474 |
| cg20701182 | 0.400 | TRUE | 0.304 | 0.325 |
| cg03142586 | 0.400 | NA | NA | 0.453 |
| cg09274094 | 0.399 | NA | NA | 0.372 |
| cg13564825 | 0.398 | TRUE | 0.442 | 0.467 |
| cg07530798 | 0.395 | NA | NA | NA |
| cg11706983 | 0.395 | TRUE | 0.449 | 0.413 |
| cg04823311 | 0.394 | TRUE | 0.436 | 0.476 |
| cg11595545 | 0.394 | TRUE | 0.429 | 0.475 |
| cg06646705 | 0.392 | NA | NA | NA |
| cg20884887 | 0.391 | TRUE | 0.453 | 0.434 |
| cg13717446 | 0.391 | TRUE | 0.343 | 0.449 |
| cg14511739 | 0.389 | TRUE | 0.418 | 0.501 |
| cg07783282 | 0.389 | TRUE | 0.382 | 0.361 |
| cg27452341 | 0.389 | TRUE | 0.337 | 0.371 |
| cg00672689 | 0.388 | NA | NA | NA |
| cg14479889 | 0.388 | TRUE | 0.260 | 0.367 |
| cg14263942 | 0.387 | TRUE | 0.371 | 0.404 |
| cg14776962 | 0.387 | TRUE | 0.379 | 0.326 |
| cg17736443 | 0.387 | TRUE | 0.468 | 0.458 |
| cg07689503 | 0.386 | TRUE | 0.356 | 0.459 |
| cg19669107 | 0.386 | NA | NA | NA |
| cg19250799 | 0.386 | TRUE | 0.332 | 0.408 |
| cg21890726 | 0.385 | TRUE | 0.385 | 0.417 |
| cg19698668 | 0.385 | TRUE | 0.338 | 0.370 |
| cg18110168 | 0.384 | TRUE | 0.343 | 0.368 |
| cg22348673 | 0.384 | TRUE | 0.291 | 0.332 |
| cg14979301 | 0.384 | NA | NA | 0.414 |
| cg00577935 | 0.383 | TRUE | 0.330 | 0.416 |
| cg14861089 | 0.383 | TRUE | 0.442 | 0.463 |
| cg03683587 | 0.383 | NA | NA | 0.378 |
| cg10119082 | 0.383 | TRUE | 0.319 | 0.418 |
| cg15457058 | 0.383 | TRUE | 0.346 | 0.448 |
| cg12640394 | 0.383 | NA | NA | 0.430 |
| cg12840719 | 0.382 | NA | NA | 0.422 |
| cg06382344 | 0.382 | TRUE | 0.402 | 0.438 |
| cg25214789 | 0.381 | TRUE | 0.353 | 0.373 |
| cg02736548 | 0.380 | NA | NA | 0.441 |
| cg16601494 | 0.379 | TRUE | 0.417 | 0.437 |
| cg10376598 | 0.379 | TRUE | 0.314 | 0.406 |
| cg00817367 | 0.379 | TRUE | 0.482 | 0.501 |
| cg06537894 | 0.378 | NA | NA | 0.470 |
| cg24432073 | 0.378 | TRUE | 0.322 | 0.340 |
| cg17300544 | 0.377 | TRUE | 0.428 | 0.563 |
| cg20261859 | 0.377 | NA | NA | NA |
| cg17014785 | 0.377 | TRUE | 0.322 | 0.308 |
| cg12783819 | 0.377 | NA | NA | 0.492 |
| cg07124687 | 0.377 | TRUE | 0.490 | 0.434 |
| cg21790626 | 0.376 | TRUE | 0.463 | 0.474 |
| cg03918605 | 0.376 | NA | NA | NA |
| cg06991484 | 0.376 | TRUE | 0.367 | 0.339 |
| cg06829686 | 0.376 | TRUE | 0.454 | 0.443 |
| cg26618965 | 0.375 | NA | NA | 0.440 |
| cg23391785 | 0.375 | TRUE | 0.418 | 0.429 |
| cg14249876 | 0.374 | TRUE | 0.295 | 0.362 |
| cg00256076 | 0.374 | NA | NA | NA |
| cg17154724 | 0.373 | NA | NA | 0.509 |
| cg00970396 | 0.372 | TRUE | 0.461 | 0.416 |
| cg13759674 | 0.372 | TRUE | 0.425 | 0.448 |
| cg00922376 | 0.372 | TRUE | 0.397 | 0.425 |
| cg23982858 | 0.372 | TRUE | 0.307 | 0.315 |
| cg10659805 | 0.372 | TRUE | 0.424 | 0.437 |
| cg16579555 | 0.371 | TRUE | 0.342 | 0.396 |
| cg00033551 | 0.370 | TRUE | 0.528 | 0.443 |
| cg25622366 | 0.369 | TRUE | 0.497 | 0.480 |
| cg25668172 | 0.369 | NA | NA | NA |
| cg00753478 | 0.369 | TRUE | 0.400 | 0.387 |
| cg12260798 | 0.369 | TRUE | 0.314 | 0.246 |
| cg05554189 | 0.368 | NA | NA | NA |
| cg04845566 | 0.368 | NA | NA | NA |
| cg18470455 | 0.368 | TRUE | 0.263 | 0.310 |
| cg27234864 | 0.367 | TRUE | 0.399 | 0.392 |
| cg12451631 | 0.367 | NA | NA | 0.389 |
| cg26375010 | 0.367 | TRUE | 0.327 | 0.242 |
| cg25340966 | 0.366 | TRUE | 0.414 | 0.359 |
| cg27085741 | 0.366 | TRUE | 0.384 | 0.415 |
| cg12206199 | 0.366 | TRUE | 0.376 | 0.385 |
| cg14310034 | 0.366 | TRUE | 0.344 | 0.341 |
| cg00036011 | 0.366 | TRUE | 0.248 | 0.283 |
| cg17436134 | 0.365 | NA | NA | 0.237 |
| cg08668790 | 0.365 | TRUE | 0.382 | 0.379 |
| cg04819760 | 0.365 | TRUE | 0.239 | 0.271 |
| cg11672054 | 0.365 | TRUE | 0.302 | 0.332 |
| cg22884656 | 0.364 | NA | NA | 0.389 |
| cg08571859 | 0.364 | TRUE | 0.300 | 0.374 |
| cg00927554 | 0.362 | NA | NA | 0.304 |
| cg25524962 | 0.362 | TRUE | 0.299 | 0.375 |
| cg27049766 | 0.362 | TRUE | 0.389 | 0.402 |
| cg00983956 | 0.361 | NA | NA | NA |
| cg14159026 | 0.361 | TRUE | 0.402 | 0.429 |
| cg11284811 | 0.361 | NA | NA | NA |
| cg10397765 | 0.361 | TRUE | 0.389 | 0.353 |
| cg19302722 | 0.361 | TRUE | 0.304 | 0.258 |
| cg10703826 | 0.361 | TRUE | 0.488 | 0.427 |
| cg14988503 | 0.360 | TRUE | 0.334 | 0.328 |
| cg08566455 | 0.360 | NA | NA | 0.488 |
| cg05319845 | 0.360 | NA | NA | NA |
| cg15991309 | 0.360 | TRUE | 0.287 | 0.354 |
| cg15471815 | 0.359 | TRUE | 0.278 | 0.324 |
| cg05488523 | 0.359 | TRUE | 0.252 | 0.307 |
| cg13096208 | 0.359 | TRUE | 0.468 | 0.468 |
| cg24918608 | 0.359 | NA | NA | NA |
| cg17825572 | 0.359 | TRUE | 0.353 | 0.317 |
| cg02659794 | 0.359 | TRUE | 0.281 | 0.307 |
| cg01108370 | 0.358 | TRUE | 0.268 | 0.330 |
| cg07961512 | 0.358 | NA | NA | 0.330 |
| cg25767001 | 0.358 | NA | NA | NA |
| cg02858118 | 0.358 | TRUE | 0.314 | 0.331 |
| cg18582180 | 0.358 | TRUE | 0.427 | 0.449 |
| cg16042259 | 0.358 | TRUE | 0.333 | 0.317 |
| cg05152874 | 0.357 | TRUE | 0.375 | 0.387 |
| cg24416513 | 0.357 | TRUE | 0.434 | 0.457 |
| cg07311841 | 0.357 | NA | NA | NA |
| cg18555909 | 0.356 | NA | NA | NA |
| cg17655851 | 0.356 | NA | NA | NA |
| cg04039555 | 0.356 | NA | NA | 0.321 |
| cg02864844 | 0.356 | NA | NA | 0.434 |
| cg16977637 | 0.356 | NA | NA | NA |
| cg04167595 | 0.356 | TRUE | 0.241 | 0.240 |
| cg13394153 | 0.356 | NA | NA | NA |
| cg00948275 | 0.355 | NA | NA | 0.378 |
| cg01121566 | 0.355 | NA | NA | NA |
| cg17689712 | 0.355 | NA | NA | NA |
| cg20971220 | 0.355 | TRUE | 0.252 | 0.295 |
| cg21063722 | 0.355 | NA | NA | 0.417 |
| cg12710510 | 0.354 | TRUE | 0.234 | 0.313 |
| cg25330243 | 0.354 | TRUE | 0.351 | 0.295 |
| cg17204129 | 0.354 | TRUE | 0.275 | 0.314 |
| cg22796507 | 0.354 | NA | NA | 0.455 |
| cg23657560 | 0.354 | NA | NA | NA |
| cg20449590 | 0.354 | NA | NA | 0.501 |
| cg19746780 | 0.354 | TRUE | 0.250 | 0.233 |
| cg05379541 | 0.353 | TRUE | 0.374 | 0.341 |
| cg19809499 | 0.353 | TRUE | 0.397 | 0.483 |
| cg10648670 | 0.353 | NA | NA | 0.368 |
| cg13176741 | 0.353 | TRUE | 0.294 | 0.299 |
| cg12945444 | 0.353 | TRUE | 0.355 | 0.306 |
| cg22447539 | 0.353 | TRUE | 0.336 | 0.403 |
| cg17297071 | 0.353 | TRUE | 0.337 | 0.342 |
| cg11731890 | 0.352 | TRUE | 0.292 | 0.312 |
| cg08571241 | 0.352 | NA | NA | NA |
| cg16561266 | 0.352 | TRUE | 0.317 | 0.410 |
| cg16306898 | 0.352 | NA | NA | 0.462 |
| cg19306047 | 0.351 | TRUE | 0.362 | 0.455 |
| cg27583690 | 0.351 | NA | NA | 0.407 |
| cg03621881 | 0.351 | NA | NA | 0.502 |
| cg20399509 | 0.350 | TRUE | 0.348 | 0.377 |
| cg04515996 | 0.350 | TRUE | 0.304 | 0.315 |
| cg09098624 | 0.350 | TRUE | 0.221 | 0.272 |
| cg04034767 | 0.350 | NA | NA | 0.489 |
| cg18932798 | 0.350 | TRUE | 0.418 | 0.422 |
| cg27636310 | 0.350 | TRUE | 0.334 | 0.302 |
| cg18623980 | 0.349 | TRUE | 0.425 | 0.395 |
| cg26438317 | 0.349 | NA | NA | NA |
| cg19389884 | 0.349 | TRUE | 0.277 | 0.303 |
| cg06531379 | 0.348 | TRUE | 0.375 | 0.372 |
| cg13997680 | 0.348 | NA | NA | NA |
| cg13481969 | 0.348 | TRUE | 0.465 | 0.451 |
| cg15293367 | 0.348 | NA | NA | NA |
| cg24505678 | 0.348 | NA | NA | NA |
| cg22510376 | 0.348 | NA | NA | 0.413 |
| cg08154437 | 0.347 | TRUE | 0.435 | 0.483 |
| cg26010734 | 0.347 | NA | NA | 0.460 |
| cg22524061 | 0.347 | TRUE | 0.563 | 0.498 |
| cg26005578 | 0.347 | TRUE | 0.327 | 0.351 |
| cg23433889 | 0.347 | TRUE | 0.229 | 0.324 |
| cg00573330 | 0.347 | TRUE | 0.322 | 0.381 |
| cg16317734 | 0.347 | TRUE | 0.202 | 0.263 |
| cg01581084 | 0.347 | TRUE | 0.356 | 0.371 |
| cg07779120 | 0.347 | TRUE | 0.497 | 0.443 |
| cg21176475 | 0.347 | TRUE | 0.277 | 0.378 |
| cg19706516 | 0.346 | TRUE | 0.226 | 0.265 |
| cg14273822 | 0.346 | TRUE | 0.263 | 0.291 |
| cg27639620 | 0.346 | NA | NA | 0.336 |
| cg23923856 | 0.346 | TRUE | 0.306 | 0.383 |
| cg00773413 | 0.346 | TRUE | 0.365 | 0.389 |
| cg20275528 | 0.346 | TRUE | 0.367 | 0.478 |
| cg25480336 | 0.346 | TRUE | 0.384 | 0.407 |
| cg19499748 | 0.346 | TRUE | 0.228 | 0.270 |
| cg07381000 | 0.346 | TRUE | 0.324 | 0.262 |
| cg04813832 | 0.346 | NA | NA | NA |
| cg23634087 | 0.345 | TRUE | 0.314 | 0.409 |
| cg11766986 | 0.345 | NA | NA | NA |
| cg17003293 | 0.345 | TRUE | 0.337 | 0.382 |
| cg22158769 | 0.345 | TRUE | 0.371 | 0.368 |
| cg24722073 | 0.344 | TRUE | 0.366 | 0.354 |
| cg18732064 | 0.344 | TRUE | 0.317 | 0.368 |
| cg01204606 | 0.344 | TRUE | 0.292 | 0.280 |
| cg25720804 | 0.344 | TRUE | 0.395 | 0.370 |
| cg05661282 | 0.344 | TRUE | 0.414 | 0.417 |
| cg21572621 | 0.344 | TRUE | 0.220 | 0.276 |
| cg08160350 | 0.344 | TRUE | 0.288 | 0.339 |
| cg02466113 | 0.344 | TRUE | 0.267 | 0.301 |
| cg14175690 | 0.343 | NA | NA | 0.397 |
| cg24393316 | 0.343 | TRUE | 0.347 | 0.356 |
| cg11294513 | 0.343 | TRUE | 0.349 | 0.371 |
| cg02320862 | 0.343 | TRUE | 0.350 | 0.453 |
| cg27398263 | 0.343 | TRUE | 0.316 | 0.377 |
| cg06594803 | 0.343 | NA | NA | NA |
| cg18756179 | 0.343 | TRUE | 0.410 | 0.444 |
| cg08657361 | 0.343 | TRUE | 0.173 | 0.253 |
| cg05804220 | 0.343 | TRUE | 0.426 | 0.403 |
| cg03394725 | 0.343 | NA | NA | NA |
| cg05191879 | 0.343 | NA | NA | 0.365 |
| cg26989048 | 0.343 | NA | NA | NA |
| cg18854166 | 0.342 | NA | NA | NA |
| cg16408715 | 0.342 | NA | NA | NA |
| cg25945732 | 0.342 | TRUE | 0.340 | 0.476 |
| cg18202336 | 0.342 | TRUE | 0.281 | 0.335 |
| cg03585419 | 0.342 | TRUE | 0.322 | 0.307 |
| cg02769145 | 0.342 | TRUE | 0.295 | 0.284 |
| cg09349604 | 0.342 | TRUE | 0.315 | 0.349 |
| cg04809136 | 0.342 | TRUE | 0.251 | 0.311 |
| cg20486569 | 0.342 | TRUE | 0.335 | 0.341 |
| cg14315558 | 0.342 | TRUE | 0.344 | 0.350 |
| cg13185030 | 0.341 | TRUE | 0.296 | 0.421 |
| cg11032268 | 0.341 | NA | NA | 0.462 |
| cg03308628 | 0.341 | TRUE | 0.278 | 0.318 |
| cg20980783 | 0.341 | TRUE | 0.441 | 0.435 |
| cg20232986 | 0.341 | TRUE | 0.327 | 0.352 |
| cg11957331 | 0.341 | TRUE | 0.305 | 0.291 |
| cg02408775 | 0.341 | TRUE | 0.257 | 0.337 |
| cg02659086 | 0.341 | TRUE | 0.247 | 0.211 |
| cg17445666 | 0.341 | NA | NA | NA |
| cg10583297 | 0.341 | TRUE | 0.343 | 0.307 |
| cg18348142 | 0.341 | NA | NA | 0.383 |
| cg08686553 | 0.341 | NA | NA | NA |
| cg23616139 | 0.341 | TRUE | 0.190 | 0.279 |
| cg16358679 | 0.340 | TRUE | 0.353 | 0.389 |
| cg22806907 | 0.340 | TRUE | 0.310 | 0.326 |
| cg04550737 | 0.340 | TRUE | 0.347 | 0.378 |
| cg01098320 | 0.340 | NA | NA | 0.304 |
| cg00108164 | 0.340 | TRUE | 0.323 | 0.463 |
| cg21472506 | 0.340 | TRUE | 0.500 | 0.459 |
| cg11756095 | 0.340 | TRUE | 0.356 | 0.382 |
| cg09187633 | 0.340 | NA | NA | NA |
| cg20749741 | 0.340 | TRUE | 0.309 | 0.460 |
| cg23337116 | 0.339 | TRUE | 0.327 | 0.408 |
| cg22750156 | 0.339 | NA | NA | NA |
| cg21301805 | 0.339 | TRUE | 0.345 | 0.323 |
| cg21265540 | 0.339 | TRUE | 0.338 | 0.442 |
| cg25011396 | 0.339 | NA | NA | NA |
| cg15798385 | 0.339 | TRUE | 0.364 | 0.331 |
| cg01558212 | 0.339 | TRUE | 0.302 | 0.331 |
| cg00501628 | 0.339 | TRUE | 0.183 | 0.171 |
| cg14209005 | 0.339 | TRUE | 0.247 | 0.248 |
| cg03234186 | 0.338 | TRUE | 0.313 | 0.315 |
| cg21107197 | 0.338 | TRUE | 0.347 | 0.353 |
| cg12687570 | 0.338 | NA | NA | NA |
| cg19270505 | 0.338 | NA | NA | 0.373 |
| cg09548780 | 0.338 | TRUE | 0.374 | 0.295 |
| cg18786593 | 0.338 | TRUE | 0.407 | 0.340 |
| cg20129213 | 0.338 | TRUE | 0.428 | 0.476 |
| cg12506930 | 0.338 | TRUE | 0.304 | 0.320 |
| cg16966315 | 0.338 | TRUE | 0.348 | 0.306 |
| cg25344734 | 0.338 | TRUE | 0.217 | 0.257 |
| cg21332500 | 0.337 | TRUE | 0.338 | 0.318 |
| cg02168303 | 0.337 | TRUE | 0.342 | 0.367 |
| cg20885782 | 0.337 | TRUE | 0.304 | 0.335 |
| cg26440289 | 0.337 | TRUE | 0.465 | 0.474 |
| cg22789900 | 0.337 | TRUE | 0.386 | 0.463 |
| cg00716257 | 0.337 | TRUE | 0.329 | 0.311 |
| cg03468349 | 0.336 | TRUE | 0.370 | 0.489 |
| cg01786994 | 0.336 | NA | NA | 0.424 |
| cg05068987 | 0.336 | TRUE | 0.299 | 0.319 |
| cg13877670 | 0.336 | TRUE | 0.236 | 0.297 |
| cg05145435 | 0.336 | NA | NA | NA |
| cg01871214 | 0.336 | NA | NA | 0.363 |
| cg18153401 | 0.336 | NA | NA | NA |
| cg11173146 | 0.336 | TRUE | 0.364 | 0.456 |
| cg22568351 | 0.336 | NA | NA | NA |
| cg10171448 | 0.336 | TRUE | 0.440 | 0.443 |
| cg16970232 | 0.336 | TRUE | 0.238 | 0.351 |
| cg08814148 | 0.336 | TRUE | 0.326 | 0.293 |
| cg15544654 | 0.335 | TRUE | 0.308 | 0.309 |
| cg08167706 | 0.335 | TRUE | 0.329 | 0.351 |
| cg08742288 | 0.335 | TRUE | 0.343 | 0.411 |
| cg10483825 | 0.335 | TRUE | 0.230 | 0.245 |
| cg15732502 | 0.335 | TRUE | 0.316 | 0.340 |
| cg03111404 | 0.335 | NA | NA | 0.394 |
| cg02161084 | 0.334 | NA | NA | 0.381 |
| cg26635219 | 0.334 | TRUE | 0.381 | 0.417 |
| cg12172441 | 0.334 | NA | NA | 0.357 |
| cg16097357 | 0.334 | TRUE | 0.284 | 0.345 |
| cg16701896 | 0.334 | NA | NA | 0.380 |
| cg26117023 | 0.334 | TRUE | 0.293 | 0.336 |
| cg09260089 | 0.334 | TRUE | 0.360 | 0.364 |
| cg14931854 | 0.333 | TRUE | 0.427 | 0.418 |
| cg22534203 | 0.333 | NA | NA | NA |
| cg10496082 | 0.333 | TRUE | 0.356 | 0.314 |
| cg25324047 | 0.333 | TRUE | 0.358 | 0.350 |
| cg19729116 | 0.333 | TRUE | 0.273 | 0.349 |
| cg15487867 | 0.333 | NA | NA | 0.477 |
| cg05196969 | 0.333 | TRUE | 0.337 | 0.349 |
| cg02755525 | 0.333 | TRUE | 0.311 | 0.398 |
| cg03437479 | 0.333 | TRUE | 0.254 | 0.297 |
| cg25905674 | 0.333 | TRUE | 0.326 | 0.390 |
| cg02373152 | 0.332 | TRUE | 0.282 | 0.358 |
| cg06180621 | 0.332 | NA | NA | NA |
| cg16980360 | 0.332 | TRUE | 0.242 | 0.270 |
| cg24292235 | 0.332 | TRUE | 0.244 | 0.297 |
| cg23559228 | 0.332 | NA | NA | NA |
| cg24948962 | 0.332 | TRUE | 0.242 | 0.309 |
| cg04786857 | 0.332 | TRUE | 0.341 | 0.295 |
| cg12177220 | 0.332 | TRUE | 0.276 | 0.275 |
| cg00527440 | 0.332 | TRUE | 0.288 | 0.380 |
| cg12136608 | 0.332 | TRUE | 0.233 | 0.274 |
| cg04152056 | 0.331 | NA | NA | NA |
| cg05237485 | 0.331 | TRUE | 0.223 | 0.233 |
| cg08331374 | 0.331 | NA | NA | NA |
| cg13850606 | 0.331 | TRUE | 0.348 | 0.331 |
| cg05413277 | 0.331 | TRUE | 0.184 | 0.214 |
| cg23838378 | 0.331 | NA | NA | NA |
| cg26674751 | 0.331 | NA | NA | NA |
| cg01563704 | 0.331 | TRUE | 0.290 | 0.304 |
| cg20401551 | 0.331 | TRUE | 0.218 | 0.314 |
| cg07119434 | 0.330 | TRUE | 0.184 | 0.255 |
| cg18514820 | 0.330 | TRUE | 0.312 | 0.355 |
| cg04088969 | 0.330 | TRUE | 0.281 | 0.306 |
| cg16182707 | 0.330 | NA | NA | 0.364 |
| cg04849842 | 0.330 | NA | NA | 0.311 |
| cg21170796 | 0.330 | NA | NA | 0.212 |
| cg04021697 | 0.330 | TRUE | 0.398 | 0.451 |
| cg01362570 | 0.330 | TRUE | 0.218 | 0.304 |
| cg10799055 | 0.329 | TRUE | 0.363 | 0.297 |
| cg00656387 | 0.329 | TRUE | 0.230 | 0.267 |
| cg02831587 | 0.329 | TRUE | 0.312 | 0.302 |
| cg22497741 | 0.329 | NA | NA | 0.438 |
| cg18150909 | 0.329 | NA | NA | NA |
| cg23371746 | 0.329 | TRUE | 0.358 | 0.344 |
| cg10168149 | 0.329 | TRUE | 0.367 | 0.416 |
| cg24724633 | 0.329 | TRUE | 0.267 | 0.352 |
| cg06470822 | 0.329 | TRUE | 0.336 | 0.332 |
| cg01120173 | 0.328 | TRUE | 0.342 | 0.319 |
| cg13409248 | 0.328 | TRUE | 0.283 | 0.285 |
| cg17721249 | 0.328 | NA | NA | 0.325 |
| cg26236177 | 0.328 | TRUE | 0.349 | 0.324 |
| cg05998607 | 0.328 | NA | NA | 0.216 |
| cg14032025 | 0.328 | NA | NA | 0.465 |
| cg10272601 | 0.328 | TRUE | 0.347 | 0.372 |
| cg23246885 | 0.328 | TRUE | 0.420 | 0.486 |
| cg19980771 | 0.328 | NA | NA | 0.364 |
| cg06378871 | 0.328 | NA | NA | 0.412 |
| cg17569842 | 0.327 | TRUE | 0.488 | 0.425 |
| cg17172980 | 0.327 | TRUE | 0.354 | 0.405 |
| cg17246382 | 0.327 | TRUE | 0.375 | 0.449 |
| cg08558397 | 0.327 | TRUE | 0.332 | 0.401 |
| cg13530938 | 0.327 | TRUE | 0.348 | 0.344 |
| cg17616554 | 0.327 | TRUE | 0.295 | 0.315 |
| cg16531209 | 0.327 | NA | NA | 0.261 |
| cg22592142 | 0.327 | TRUE | 0.317 | 0.294 |
| cg10363885 | 0.327 | TRUE | 0.146 | 0.119 |
| cg01964152 | 0.327 | TRUE | 0.279 | 0.260 |
| cg12680609 | 0.327 | NA | NA | NA |
| cg22352818 | 0.327 | TRUE | 0.321 | 0.338 |
| cg24770624 | 0.326 | TRUE | 0.197 | 0.259 |
| cg06793562 | 0.326 | TRUE | 0.366 | 0.324 |
| cg08460435 | 0.326 | TRUE | 0.295 | 0.305 |
| cg05860723 | 0.326 | TRUE | 0.421 | 0.412 |
| cg08584377 | 0.326 | NA | NA | 0.496 |
| cg22531183 | 0.326 | TRUE | 0.394 | 0.455 |
| cg16990168 | 0.326 | TRUE | 0.324 | 0.320 |
| cg19852958 | 0.326 | TRUE | 0.456 | 0.475 |
| cg08955613 | 0.325 | NA | NA | NA |
| cg11411872 | 0.325 | NA | NA | NA |
| cg04907523 | 0.325 | TRUE | 0.345 | 0.370 |
| cg04645914 | 0.325 | TRUE | 0.289 | 0.301 |
| cg22571664 | 0.325 | TRUE | 0.234 | 0.259 |
| cg19872036 | 0.325 | NA | NA | NA |
| cg06392169 | 0.325 | TRUE | 0.326 | 0.365 |
| cg01528052 | 0.325 | TRUE | 0.409 | 0.359 |
| cg03774463 | 0.325 | TRUE | 0.326 | 0.325 |
| cg07274590 | 0.325 | TRUE | 0.258 | 0.209 |
| cg11940285 | 0.325 | TRUE | 0.381 | 0.318 |
| cg12642693 | 0.325 | TRUE | 0.226 | 0.248 |
| cg03306486 | 0.325 | TRUE | 0.358 | 0.496 |
| cg08865099 | 0.325 | TRUE | 0.343 | 0.324 |
| cg22740547 | 0.325 | TRUE | 0.334 | 0.415 |
| cg21185289 | 0.325 | TRUE | 0.314 | 0.351 |
| cg12600174 | 0.324 | NA | NA | 0.359 |
| cg02362103 | 0.324 | TRUE | 0.333 | 0.306 |
| cg19337852 | 0.324 | TRUE | 0.325 | 0.340 |
| cg06970510 | 0.324 | TRUE | 0.327 | 0.353 |
| cg00701692 | 0.324 | TRUE | 0.296 | 0.293 |
| cg04489012 | 0.324 | TRUE | 0.265 | 0.304 |
| cg13936452 | 0.324 | TRUE | 0.416 | 0.396 |
| cg10716835 | 0.324 | TRUE | 0.242 | 0.274 |
| cg14605874 | 0.324 | TRUE | 0.301 | 0.341 |
| cg16174370 | 0.324 | NA | NA | NA |
| cg02578368 | 0.324 | TRUE | 0.346 | 0.452 |
| cg00338735 | 0.324 | TRUE | 0.263 | 0.392 |
| cg24884142 | 0.324 | TRUE | 0.371 | 0.331 |
| cg15514087 | 0.323 | NA | NA | NA |
| cg12296772 | 0.323 | NA | NA | 0.400 |
| cg23676151 | 0.323 | TRUE | 0.342 | 0.325 |
| cg10281741 | 0.323 | NA | NA | NA |
| cg18456523 | 0.323 | TRUE | 0.324 | 0.328 |
| cg02743713 | 0.323 | TRUE | 0.326 | 0.290 |
| cg02776659 | 0.323 | TRUE | 0.283 | 0.294 |
| cg02038168 | 0.323 | TRUE | 0.299 | 0.393 |
| cg24957851 | 0.323 | NA | NA | NA |
| cg25810247 | 0.323 | NA | NA | 0.327 |
| cg21416498 | 0.323 | NA | NA | NA |
| cg08703872 | 0.323 | TRUE | 0.305 | 0.344 |
| cg08081156 | 0.323 | TRUE | 0.396 | 0.411 |
| cg13878010 | 0.323 | TRUE | 0.253 | 0.323 |
| cg22488797 | 0.323 | TRUE | 0.373 | 0.330 |
| cg24425021 | 0.323 | TRUE | 0.279 | 0.350 |
| cg20399616 | 0.323 | TRUE | 0.437 | 0.428 |
| cg09557387 | 0.323 | NA | NA | 0.379 |
| cg18794404 | 0.323 | TRUE | 0.368 | 0.437 |
| cg11176990 | 0.322 | TRUE | 0.292 | 0.300 |
| cg19380001 | 0.322 | TRUE | 0.233 | 0.298 |
| cg21762523 | 0.322 | TRUE | 0.336 | 0.351 |
| cg05904135 | 0.322 | TRUE | 0.382 | 0.419 |
| cg07279070 | 0.322 | TRUE | 0.255 | 0.277 |
| cg10276465 | 0.322 | TRUE | 0.200 | 0.293 |
| cg24659054 | 0.322 | TRUE | 0.371 | 0.463 |
| cg06893138 | 0.322 | TRUE | 0.247 | 0.252 |
| cg04012924 | 0.322 | TRUE | 0.281 | 0.296 |
| cg15680620 | 0.322 | TRUE | 0.237 | 0.218 |
| cg05158615 | 0.322 | TRUE | 0.275 | 0.282 |
| cg08100565 | 0.322 | TRUE | 0.338 | 0.438 |
| cg18210732 | 0.322 | TRUE | 0.299 | 0.371 |
| cg26468478 | 0.322 | TRUE | 0.302 | 0.329 |
| cg00436476 | 0.322 | TRUE | 0.299 | 0.214 |
| cg14353137 | 0.322 | TRUE | 0.425 | 0.448 |
| cg02909790 | 0.322 | TRUE | 0.211 | 0.260 |
| cg00949442 | 0.322 | NA | NA | NA |
| cg07835424 | 0.322 | TRUE | 0.407 | 0.455 |
| cg17386213 | 0.322 | TRUE | 0.416 | 0.343 |
| cg20952599 | 0.321 | NA | NA | NA |
| cg13314979 | 0.321 | TRUE | 0.125 | 0.173 |
| cg03685124 | 0.321 | NA | NA | NA |
| cg02766845 | 0.321 | NA | NA | 0.332 |
| cg16620382 | 0.321 | TRUE | 0.392 | 0.407 |
| cg22557662 | 0.321 | TRUE | 0.430 | 0.450 |
| cg03942051 | 0.321 | TRUE | 0.218 | 0.200 |
| cg14732324 | 0.321 | TRUE | 0.391 | 0.426 |
| cg01447112 | 0.321 | TRUE | 0.383 | 0.412 |
| cg06848185 | 0.321 | TRUE | 0.349 | 0.432 |
| cg00334821 | 0.321 | TRUE | 0.175 | 0.250 |
| cg06333957 | 0.321 | NA | NA | NA |
| cg08304190 | 0.320 | TRUE | 0.454 | 0.450 |
| cg21647227 | 0.320 | TRUE | 0.277 | 0.258 |
| cg24399712 | 0.320 | TRUE | 0.204 | 0.253 |
| cg01972751 | 0.320 | NA | NA | 0.381 |
| cg04148762 | 0.320 | TRUE | 0.258 | 0.301 |
| cg24657817 | 0.320 | TRUE | 0.367 | 0.355 |
| cg21016778 | 0.320 | NA | NA | NA |
| cg12659883 | 0.320 | TRUE | 0.299 | 0.318 |
| cg06428055 | 0.320 | TRUE | 0.207 | 0.257 |
| cg25026529 | 0.320 | TRUE | 0.352 | 0.395 |
| cg24425838 | 0.320 | TRUE | 0.292 | 0.308 |
| cg18803104 | 0.320 | TRUE | 0.459 | 0.410 |
| cg18944010 | 0.319 | TRUE | 0.313 | 0.328 |
| cg05457696 | 0.319 | NA | NA | NA |
| cg26149244 | 0.319 | TRUE | 0.446 | 0.439 |
| cg01148781 | 0.319 | TRUE | 0.320 | 0.300 |
| cg20150591 | 0.319 | TRUE | 0.305 | 0.385 |
| cg27364741 | 0.319 | TRUE | 0.443 | 0.418 |
| cg09398550 | 0.319 | TRUE | 0.328 | 0.359 |
| cg24853724 | 0.319 | TRUE | 0.234 | 0.285 |
| cg07455954 | 0.319 | NA | NA | NA |
| cg10695848 | 0.319 | TRUE | 0.189 | 0.251 |
| cg16924337 | 0.319 | TRUE | 0.385 | 0.405 |
| cg11914744 | 0.319 | NA | NA | NA |
| cg08940787 | 0.319 | TRUE | 0.223 | 0.299 |
| cg22773661 | 0.319 | NA | NA | 0.418 |
| cg16058493 | 0.319 | NA | NA | 0.338 |
| cg21027189 | 0.319 | NA | NA | NA |
| cg07042902 | 0.319 | NA | NA | NA |
| cg27232866 | 0.319 | TRUE | 0.320 | 0.350 |
| cg15204206 | 0.319 | NA | NA | NA |
| cg21051519 | 0.319 | TRUE | 0.369 | 0.308 |
| cg00458878 | 0.319 | TRUE | 0.352 | 0.345 |
| cg10243939 | 0.318 | TRUE | 0.231 | 0.208 |
| cg25720795 | 0.318 | TRUE | 0.205 | 0.245 |
| cg14858267 | 0.318 | NA | NA | 0.298 |
| cg23049458 | 0.318 | TRUE | 0.392 | 0.399 |
| cg24947764 | 0.318 | TRUE | 0.347 | 0.387 |
| cg10755058 | 0.318 | TRUE | 0.291 | 0.306 |
| cg10143811 | 0.318 | TRUE | 0.338 | 0.322 |
| cg24842086 | 0.318 | TRUE | 0.266 | 0.238 |
| cg23979458 | 0.318 | NA | NA | 0.364 |
| cg26460092 | 0.318 | TRUE | 0.369 | 0.405 |
| cg25947878 | 0.318 | TRUE | 0.265 | 0.318 |
| cg06818605 | 0.318 | TRUE | 0.247 | 0.290 |
| cg17083494 | 0.318 | TRUE | 0.229 | 0.183 |
| cg01240444 | 0.318 | TRUE | 0.226 | 0.357 |
| cg18161327 | 0.318 | TRUE | 0.403 | 0.352 |
| cg24035245 | 0.318 | TRUE | 0.435 | 0.439 |
| cg08863953 | 0.318 | TRUE | 0.265 | 0.267 |
| cg09994356 | 0.318 | TRUE | 0.371 | 0.426 |
| cg14542554 | 0.318 | NA | NA | 0.247 |
| cg25200152 | 0.318 | NA | NA | 0.328 |
| cg08876434 | 0.317 | TRUE | 0.324 | 0.391 |
| cg13545212 | 0.317 | TRUE | 0.398 | 0.355 |
| cg01268824 | 0.317 | TRUE | 0.435 | 0.391 |
| cg25255293 | 0.317 | TRUE | 0.335 | 0.316 |
| cg14540778 | 0.317 | NA | NA | NA |
| cg12353688 | 0.317 | TRUE | 0.293 | 0.302 |
| cg00689492 | 0.317 | TRUE | 0.286 | 0.386 |
| cg21521518 | 0.317 | TRUE | 0.298 | 0.336 |
| cg16655905 | 0.317 | TRUE | 0.271 | 0.313 |
| cg13547928 | 0.317 | NA | NA | NA |
| cg20616414 | 0.317 | TRUE | 0.296 | 0.348 |
| cg09652652 | 0.317 | TRUE | 0.370 | 0.454 |
| cg16734164 | 0.316 | TRUE | 0.259 | 0.324 |
| cg26992213 | 0.316 | TRUE | 0.304 | 0.389 |
| cg01162037 | 0.316 | NA | NA | NA |
| cg15883716 | 0.316 | TRUE | 0.273 | 0.274 |
| cg12220691 | 0.316 | TRUE | 0.240 | 0.293 |
| cg02644510 | 0.316 | TRUE | 0.388 | 0.420 |
| cg10786045 | 0.316 | NA | NA | NA |
| cg06447424 | 0.316 | NA | NA | 0.295 |
| cg00369811 | 0.316 | NA | NA | 0.319 |
| cg25508679 | 0.316 | TRUE | 0.246 | 0.281 |
| cg18118033 | 0.316 | TRUE | 0.265 | 0.245 |
| cg13784235 | 0.316 | TRUE | 0.275 | 0.283 |
| cg19497031 | 0.316 | TRUE | 0.360 | 0.359 |
| cg02863856 | 0.316 | TRUE | 0.181 | 0.268 |
| cg13647536 | 0.316 | TRUE | 0.324 | 0.303 |
| cg00891278 | 0.315 | TRUE | 0.239 | 0.241 |
| cg15504992 | 0.315 | NA | NA | NA |
| cg09524946 | 0.315 | TRUE | 0.281 | 0.259 |
| cg11950994 | 0.315 | NA | NA | NA |
| cg13080379 | 0.315 | TRUE | 0.285 | 0.293 |
| cg21453443 | 0.315 | TRUE | 0.218 | 0.301 |
| cg26465391 | 0.315 | TRUE | 0.267 | 0.321 |
| cg12307840 | 0.315 | TRUE | 0.242 | 0.316 |
| cg17716617 | 0.315 | TRUE | 0.323 | 0.401 |
| cg27323784 | 0.315 | TRUE | 0.301 | 0.273 |
| cg12874092 | 0.315 | TRUE | 0.274 | 0.289 |
| cg13895235 | 0.315 | NA | NA | 0.396 |
| cg25764899 | 0.314 | TRUE | 0.445 | 0.432 |
| cg10491410 | 0.314 | NA | NA | NA |
| cg03740978 | 0.314 | TRUE | 0.288 | 0.366 |
| cg02027945 | 0.314 | NA | NA | 0.358 |
| cg24427504 | 0.314 | TRUE | 0.264 | 0.236 |
| cg20384898 | 0.314 | TRUE | 0.274 | 0.303 |
| cg26674943 | 0.314 | TRUE | 0.341 | 0.336 |
| cg15080119 | 0.314 | TRUE | 0.242 | 0.271 |
| cg04017533 | 0.314 | TRUE | 0.277 | 0.232 |
| cg15822765 | 0.314 | TRUE | 0.407 | 0.389 |
| cg19594218 | 0.314 | TRUE | 0.249 | 0.233 |
| cg09973548 | 0.314 | NA | NA | NA |
| cg03163783 | 0.314 | TRUE | 0.315 | 0.332 |
| cg19333963 | 0.314 | TRUE | 0.336 | 0.416 |
| cg03780851 | 0.314 | TRUE | 0.286 | 0.257 |
| cg05021846 | 0.314 | TRUE | 0.287 | 0.358 |
| cg02320543 | 0.314 | TRUE | 0.263 | 0.276 |
| cg14272433 | 0.313 | NA | NA | NA |
| cg10064339 | 0.313 | TRUE | 0.359 | 0.376 |
| cg07862461 | 0.313 | TRUE | 0.244 | 0.307 |
| cg21773872 | 0.313 | TRUE | 0.408 | 0.305 |
| cg13845094 | 0.313 | TRUE | 0.277 | 0.197 |
| cg24426072 | 0.313 | TRUE | 0.367 | 0.418 |
| cg26107850 | 0.313 | TRUE | 0.285 | 0.297 |
| cg06188229 | 0.313 | TRUE | 0.299 | 0.321 |
| cg11542528 | 0.313 | NA | NA | NA |
| cg09904383 | 0.313 | TRUE | 0.316 | 0.378 |
| cg08949408 | 0.312 | TRUE | 0.281 | 0.340 |
| cg03819783 | 0.312 | TRUE | 0.259 | 0.232 |
| cg24091001 | 0.312 | NA | NA | NA |
| cg03192598 | 0.312 | TRUE | 0.477 | 0.509 |
| cg22860137 | 0.312 | TRUE | 0.181 | 0.280 |
| cg23920953 | 0.312 | TRUE | 0.396 | 0.405 |
| cg11230298 | 0.312 | NA | NA | NA |
| cg25032595 | 0.312 | TRUE | 0.458 | 0.467 |
| cg16733946 | 0.312 | TRUE | 0.245 | 0.244 |
| cg09803262 | 0.312 | TRUE | 0.293 | 0.294 |
| cg03804136 | 0.312 | TRUE | 0.343 | 0.439 |
| cg01901262 | 0.312 | NA | NA | 0.322 |
| cg20309069 | 0.312 | NA | NA | NA |
| cg03734380 | 0.312 | NA | NA | NA |
| cg02177231 | 0.311 | TRUE | 0.299 | 0.334 |
| cg08445802 | 0.311 | TRUE | 0.394 | 0.374 |
| cg06377278 | 0.311 | TRUE | 0.309 | 0.376 |
| cg02728595 | 0.311 | TRUE | 0.261 | 0.281 |
| cg20297199 | 0.311 | TRUE | 0.294 | 0.347 |
| cg11500797 | 0.311 | TRUE | 0.278 | 0.287 |
| cg07139509 | 0.311 | TRUE | 0.330 | 0.381 |
| cg00778995 | 0.311 | NA | NA | 0.222 |
| cg18267609 | 0.311 | NA | NA | NA |
| cg23305567 | 0.311 | TRUE | 0.273 | 0.248 |
| cg20803547 | 0.311 | TRUE | 0.291 | 0.334 |
| cg11856897 | 0.311 | TRUE | 0.276 | 0.298 |
| cg09195491 | 0.311 | TRUE | 0.252 | 0.195 |
| cg18430990 | 0.311 | NA | NA | NA |
| cg06989381 | 0.311 | NA | NA | NA |
| cg19741167 | 0.311 | TRUE | 0.265 | 0.295 |
| cg14250130 | 0.311 | TRUE | 0.294 | 0.398 |
| cg07610192 | 0.311 | TRUE | 0.222 | 0.262 |
| cg07396115 | 0.311 | NA | NA | NA |
| cg10182697 | 0.311 | TRUE | 0.278 | 0.315 |
| cg02553663 | 0.310 | TRUE | 0.345 | 0.396 |
| cg23817096 | 0.310 | TRUE | 0.451 | 0.454 |
| cg02018277 | 0.310 | TRUE | 0.329 | 0.338 |
| cg27621724 | 0.310 | NA | NA | NA |
| cg10959198 | 0.310 | TRUE | 0.280 | 0.263 |
| cg23623667 | 0.310 | NA | NA | 0.332 |
| cg24903183 | 0.310 | TRUE | 0.359 | 0.391 |
| cg14709691 | 0.310 | TRUE | 0.311 | 0.293 |
| cg18912855 | 0.310 | TRUE | 0.363 | 0.390 |
| cg11195259 | 0.310 | NA | NA | NA |
| cg18818834 | 0.310 | TRUE | 0.187 | 0.301 |
| cg18862481 | 0.310 | TRUE | 0.402 | 0.379 |
| cg08402365 | 0.310 | NA | NA | 0.417 |
| cg25996042 | 0.310 | NA | NA | NA |
| cg11013726 | 0.310 | TRUE | 0.207 | 0.277 |
| cg12233379 | 0.310 | TRUE | 0.242 | 0.297 |
| cg16029760 | 0.310 | TRUE | 0.236 | 0.240 |
| cg19166302 | 0.310 | TRUE | 0.234 | 0.243 |
| cg22375192 | 0.310 | TRUE | 0.401 | 0.443 |
| cg06045337 | 0.310 | TRUE | 0.287 | 0.295 |
| cg22669412 | 0.310 | NA | NA | NA |
| cg08334760 | 0.310 | NA | NA | NA |
| cg03220633 | 0.309 | TRUE | 0.348 | 0.390 |
| cg21663122 | 0.309 | TRUE | 0.314 | 0.397 |
| cg20557801 | 0.309 | TRUE | 0.245 | 0.337 |
| cg00939495 | 0.309 | TRUE | 0.358 | 0.358 |
| cg15835396 | 0.309 | TRUE | 0.161 | 0.229 |
| cg18601167 | 0.309 | NA | NA | 0.342 |
| cg21404045 | 0.309 | TRUE | 0.289 | 0.321 |
| cg02433564 | 0.309 | TRUE | 0.287 | 0.276 |
| cg24570303 | 0.309 | TRUE | 0.276 | 0.231 |
| cg01657511 | 0.309 | TRUE | 0.371 | 0.379 |
| cg04571847 | 0.309 | TRUE | 0.220 | 0.321 |
| cg10933755 | 0.309 | NA | NA | NA |
| cg00810208 | 0.309 | NA | NA | 0.273 |
| cg13791254 | 0.309 | TRUE | 0.408 | 0.382 |
| cg10599693 | 0.309 | TRUE | 0.245 | 0.263 |
| cg05040544 | 0.309 | TRUE | 0.279 | 0.398 |
| cg20383134 | 0.309 | NA | NA | NA |
| cg13674174 | 0.309 | TRUE | 0.282 | 0.303 |
| cg14644001 | 0.308 | TRUE | 0.475 | 0.424 |
| cg06563300 | 0.308 | TRUE | 0.207 | 0.214 |
| cg23095743 | 0.308 | TRUE | 0.358 | 0.388 |
| cg06215569 | 0.308 | TRUE | 0.305 | 0.309 |
| cg15467646 | 0.308 | TRUE | 0.301 | 0.288 |
| cg15044248 | 0.308 | TRUE | 0.365 | 0.466 |
| cg17610800 | 0.308 | TRUE | 0.333 | 0.299 |
| cg00735923 | 0.308 | TRUE | 0.344 | 0.326 |
| cg23167906 | 0.308 | TRUE | 0.312 | 0.317 |
| cg26506212 | 0.308 | TRUE | 0.333 | 0.323 |
| cg09626894 | 0.308 | TRUE | 0.406 | 0.426 |
| cg25884711 | 0.308 | TRUE | 0.289 | 0.293 |
| cg16489586 | 0.308 | TRUE | 0.305 | 0.305 |
| cg01961447 | 0.308 | TRUE | 0.272 | 0.332 |
| cg25076459 | 0.308 | TRUE | 0.213 | 0.225 |
| cg24274117 | 0.308 | TRUE | 0.373 | 0.428 |
| cg27212234 | 0.308 | TRUE | 0.220 | 0.235 |
| cg17930361 | 0.308 | NA | NA | 0.276 |
| cg17306261 | 0.307 | TRUE | 0.265 | 0.292 |
| cg21872764 | 0.307 | TRUE | 0.273 | 0.345 |
| cg14000000 | 0.307 | NA | NA | NA |
| cg20985450 | 0.307 | TRUE | 0.349 | 0.403 |
| cg16439564 | 0.307 | NA | NA | NA |
| cg13601799 | 0.307 | TRUE | 0.347 | 0.437 |
| cg05304543 | 0.307 | NA | NA | NA |
| cg18016826 | 0.307 | TRUE | 0.259 | 0.271 |
| cg07844931 | 0.307 | TRUE | 0.293 | 0.289 |
| cg18055610 | 0.307 | TRUE | 0.315 | 0.256 |
| cg27252696 | 0.307 | TRUE | 0.371 | 0.415 |
| cg17891101 | 0.307 | NA | NA | NA |
| cg15375239 | 0.307 | TRUE | 0.301 | 0.314 |
| cg19198568 | 0.307 | TRUE | 0.269 | 0.304 |
| cg24680586 | 0.307 | TRUE | 0.367 | 0.389 |
| cg07717632 | 0.307 | TRUE | 0.184 | 0.261 |
| cg04502985 | 0.307 | TRUE | 0.222 | 0.252 |
| cg12938338 | 0.307 | NA | NA | NA |
| cg08548396 | 0.307 | TRUE | 0.420 | 0.349 |
| cg07352345 | 0.306 | TRUE | 0.378 | 0.376 |
| cg17646820 | 0.306 | TRUE | 0.236 | 0.239 |
| cg03757145 | 0.306 | TRUE | 0.375 | 0.369 |
| cg12985929 | 0.306 | TRUE | 0.298 | 0.363 |
| cg17813946 | 0.306 | NA | NA | NA |
| cg12973941 | 0.306 | TRUE | 0.290 | 0.321 |
| cg03905847 | 0.306 | TRUE | 0.312 | 0.326 |
| cg25670330 | 0.306 | TRUE | 0.363 | 0.410 |
| cg05115157 | 0.306 | NA | NA | NA |
| cg02478448 | 0.306 | TRUE | 0.373 | 0.365 |
| cg03105222 | 0.306 | TRUE | 0.217 | 0.321 |
| cg18815943 | 0.306 | TRUE | 0.309 | 0.373 |
| cg09188099 | 0.306 | TRUE | 0.314 | 0.314 |
| cg10640072 | 0.306 | TRUE | 0.224 | 0.191 |
| cg01529637 | 0.306 | TRUE | 0.200 | 0.324 |
| cg04370314 | 0.306 | TRUE | 0.291 | 0.281 |
| cg21875802 | 0.305 | NA | NA | 0.230 |
| cg10896862 | 0.305 | TRUE | 0.296 | 0.292 |
| cg25823419 | 0.305 | TRUE | 0.290 | 0.360 |
| cg16353006 | 0.305 | TRUE | 0.252 | 0.256 |
| cg05578357 | 0.305 | NA | NA | 0.253 |
| cg13990746 | 0.305 | TRUE | 0.215 | 0.237 |
| cg20093476 | 0.305 | NA | NA | NA |
| cg21384402 | 0.305 | TRUE | 0.336 | 0.346 |
| cg21052682 | 0.305 | TRUE | 0.381 | 0.393 |
| cg04908308 | 0.305 | NA | NA | NA |
| cg14024461 | 0.305 | TRUE | 0.272 | 0.272 |
| cg21534423 | 0.305 | TRUE | 0.302 | 0.371 |
| cg17960926 | 0.305 | TRUE | 0.227 | 0.287 |
| cg14816805 | 0.305 | NA | NA | NA |
| cg26240185 | 0.305 | TRUE | 0.317 | 0.355 |
| cg04515809 | 0.304 | NA | NA | NA |
| cg20284629 | 0.304 | TRUE | 0.219 | 0.241 |
| cg00564163 | 0.304 | TRUE | 0.238 | 0.221 |
| cg25636224 | 0.304 | NA | NA | NA |
| cg19429281 | 0.304 | TRUE | 0.436 | 0.462 |
| cg23464101 | 0.304 | NA | NA | NA |
| cg02624770 | 0.304 | TRUE | 0.200 | 0.280 |
| cg20244854 | 0.304 | NA | NA | NA |
| cg04362586 | 0.304 | TRUE | 0.306 | 0.247 |
| cg25253677 | 0.304 | NA | NA | 0.377 |
| cg07104209 | 0.304 | TRUE | 0.263 | 0.270 |
| cg08640609 | 0.304 | TRUE | 0.232 | 0.392 |
| cg03420540 | 0.304 | NA | NA | NA |
| cg02746869 | 0.304 | TRUE | 0.286 | 0.320 |
| cg04336379 | 0.304 | NA | NA | 0.370 |
| cg23808946 | 0.304 | TRUE | 0.308 | 0.350 |
| cg05337743 | 0.304 | TRUE | 0.255 | 0.254 |
| cg21347053 | 0.304 | TRUE | 0.383 | 0.413 |
| cg05347898 | 0.304 | TRUE | 0.315 | 0.374 |
| cg07014523 | 0.304 | TRUE | 0.278 | 0.356 |
| cg16758800 | 0.303 | TRUE | 0.213 | 0.196 |
| cg20162652 | 0.303 | TRUE | 0.261 | 0.247 |
| cg12450224 | 0.303 | NA | NA | NA |
| cg17232003 | 0.303 | NA | NA | NA |
| cg01751057 | 0.303 | NA | NA | NA |
| cg05684891 | 0.303 | TRUE | 0.281 | 0.268 |
| cg24714905 | 0.303 | TRUE | 0.242 | 0.250 |
| cg26983469 | 0.303 | TRUE | 0.298 | 0.331 |
| cg14252279 | 0.303 | TRUE | 0.369 | 0.379 |
| cg09570682 | 0.303 | TRUE | 0.310 | 0.301 |
| cg01725199 | 0.303 | TRUE | 0.226 | 0.294 |
| cg17818432 | 0.303 | TRUE | 0.270 | 0.268 |
| cg08148261 | 0.303 | TRUE | 0.233 | 0.212 |
| cg24274579 | 0.303 | TRUE | 0.304 | 0.302 |
| cg03834338 | 0.303 | NA | NA | NA |
| cg17768491 | 0.303 | TRUE | 0.398 | 0.401 |
| cg11660826 | 0.303 | TRUE | 0.407 | 0.337 |
| cg19267596 | 0.303 | TRUE | 0.285 | 0.306 |
| cg16915821 | 0.303 | TRUE | 0.272 | 0.299 |
| cg16432061 | 0.303 | NA | NA | NA |
| cg05588623 | 0.303 | NA | NA | NA |
| cg15993383 | 0.303 | TRUE | 0.270 | 0.371 |
| cg21461649 | 0.303 | TRUE | 0.319 | 0.286 |
| cg26328510 | 0.303 | TRUE | 0.325 | 0.332 |
| cg20443254 | 0.302 | TRUE | 0.365 | 0.374 |
| cg07533148 | 0.302 | TRUE | 0.323 | 0.287 |
| cg02982294 | 0.302 | NA | NA | NA |
| cg20986370 | 0.302 | TRUE | 0.275 | 0.309 |
| cg18978493 | 0.302 | NA | NA | 0.288 |
| cg16281276 | 0.302 | TRUE | 0.396 | 0.422 |
| cg26986180 | 0.302 | TRUE | 0.209 | 0.317 |
| cg19043574 | 0.302 | TRUE | 0.276 | 0.266 |
| cg09761040 | 0.302 | TRUE | 0.271 | 0.267 |
| cg08296831 | 0.302 | TRUE | 0.303 | 0.380 |
| cg10900437 | 0.302 | TRUE | 0.267 | 0.323 |
| cg13546935 | 0.302 | TRUE | 0.390 | 0.393 |
| cg05095158 | 0.302 | TRUE | 0.341 | 0.428 |
| cg25370606 | 0.302 | NA | NA | NA |
| cg24816460 | 0.302 | TRUE | 0.376 | 0.393 |
| cg09515953 | 0.302 | TRUE | 0.368 | 0.368 |
| cg26248173 | 0.302 | TRUE | 0.327 | 0.336 |
| cg07379055 | 0.302 | TRUE | 0.309 | 0.379 |
| cg16399136 | 0.302 | TRUE | 0.205 | 0.254 |
| cg21425842 | 0.302 | TRUE | 0.348 | 0.340 |
| cg09938227 | 0.302 | TRUE | 0.288 | 0.289 |
| cg09656668 | 0.301 | NA | NA | NA |
| cg11903133 | 0.301 | TRUE | 0.355 | 0.368 |
| cg12534216 | 0.301 | TRUE | 0.306 | 0.242 |
| cg03509671 | 0.301 | TRUE | 0.247 | 0.303 |
| cg10454246 | 0.301 | TRUE | 0.216 | 0.302 |
| cg11674865 | 0.301 | NA | NA | 0.220 |
| cg25026152 | 0.301 | NA | NA | NA |
| cg19744936 | 0.301 | NA | NA | 0.320 |
| cg03929741 | 0.301 | TRUE | 0.347 | 0.407 |
| cg12382153 | 0.301 | TRUE | 0.372 | 0.338 |
| cg10572355 | 0.301 | TRUE | 0.250 | 0.320 |
| cg04595393 | 0.301 | TRUE | 0.271 | 0.249 |
| cg17975443 | 0.301 | TRUE | 0.267 | 0.288 |
| cg13900100 | 0.301 | TRUE | 0.182 | 0.205 |
| cg02784513 | 0.301 | NA | NA | NA |
| cg16437904 | 0.301 | NA | NA | NA |
| cg17236169 | 0.301 | TRUE | 0.338 | 0.301 |
| cg00251405 | 0.301 | TRUE | 0.294 | 0.398 |
| cg07605211 | 0.301 | NA | NA | 0.413 |
| cg11482719 | 0.301 | TRUE | 0.178 | 0.264 |
| cg15845792 | 0.300 | TRUE | 0.244 | 0.269 |
| cg23229820 | 0.300 | NA | NA | NA |
| cg03423149 | 0.300 | TRUE | 0.277 | 0.372 |
| cg00366850 | 0.300 | TRUE | 0.328 | 0.291 |
| cg00626110 | 0.300 | TRUE | 0.253 | 0.307 |
| cg18233405 | 0.300 | TRUE | 0.317 | 0.315 |
| cg10269548 | 0.300 | TRUE | 0.381 | 0.384 |
| cg16636355 | 0.300 | TRUE | 0.283 | 0.282 |
| cg17757602 | 0.300 | TRUE | 0.305 | 0.332 |
| cg21908638 | 0.300 | TRUE | 0.278 | 0.382 |
| cg23727983 | 0.300 | TRUE | 0.368 | 0.388 |
| cg12684209 | 0.300 | TRUE | 0.231 | 0.255 |
| cg07101909 | 0.300 | TRUE | 0.244 | 0.385 |
| cg17409893 | 0.300 | TRUE | 0.220 | 0.299 |
| cg24593688 | 0.300 | TRUE | 0.213 | 0.265 |
| cg06246094 | 0.300 | TRUE | 0.285 | 0.255 |
| cg23319982 | 0.300 | TRUE | 0.330 | 0.382 |
| cg15969216 | 0.300 | TRUE | 0.295 | 0.395 |
| cg16902385 | 0.300 | TRUE | 0.314 | 0.310 |
| cg24929737 | 0.300 | TRUE | 0.163 | 0.258 |
| cg27663938 | 0.300 | TRUE | 0.293 | 0.342 |
| cg17398252 | 0.299 | NA | NA | 0.349 |
| cg18110914 | 0.299 | NA | NA | NA |
| cg11075751 | 0.299 | NA | NA | NA |
| cg05346841 | 0.299 | TRUE | 0.329 | 0.365 |
| cg13428480 | 0.299 | TRUE | 0.359 | 0.404 |
| cg18675097 | 0.299 | TRUE | 0.344 | 0.395 |
| cg24867524 | 0.299 | TRUE | 0.170 | 0.281 |
| cg13038241 | 0.299 | NA | NA | 0.450 |
| cg09374774 | 0.299 | TRUE | 0.163 | 0.260 |
| cg19186145 | 0.299 | TRUE | 0.327 | 0.387 |
| cg03557857 | 0.299 | TRUE | 0.246 | 0.242 |
| cg06152533 | 0.299 | TRUE | 0.227 | 0.218 |
| cg26666804 | 0.299 | NA | NA | 0.367 |
| cg15661311 | 0.299 | TRUE | 0.288 | 0.327 |
| cg14866200 | 0.299 | NA | NA | 0.385 |
| cg02280532 | 0.299 | TRUE | 0.319 | 0.343 |
| cg06708634 | 0.299 | TRUE | 0.316 | 0.297 |
| cg07104660 | 0.299 | TRUE | 0.237 | 0.254 |
| cg11772171 | 0.299 | NA | NA | 0.278 |
| cg08369368 | 0.299 | TRUE | 0.323 | 0.345 |
| cg14088357 | 0.298 | TRUE | 0.306 | 0.366 |
| cg21030800 | 0.298 | NA | NA | NA |
| cg18633684 | 0.298 | TRUE | 0.211 | 0.280 |
| cg25054666 | 0.298 | NA | NA | NA |
| cg04326960 | 0.298 | NA | NA | NA |
| cg00849610 | 0.298 | TRUE | 0.271 | 0.240 |
| cg01000657 | 0.298 | NA | NA | 0.307 |
| cg14123923 | 0.298 | NA | NA | 0.318 |
| cg02008397 | 0.298 | NA | NA | NA |
| cg20232102 | 0.298 | TRUE | 0.346 | 0.403 |
| cg12781700 | 0.298 | TRUE | 0.393 | 0.399 |
| cg27181253 | 0.298 | NA | NA | 0.215 |
| cg03561565 | 0.298 | NA | NA | 0.279 |
| cg03813164 | 0.298 | TRUE | 0.295 | 0.310 |
| cg09020827 | 0.298 | NA | NA | NA |
| cg04766371 | 0.298 | TRUE | 0.219 | 0.244 |
| cg06475764 | 0.298 | TRUE | 0.175 | 0.222 |
| cg09935388 | 0.298 | TRUE | 0.251 | 0.273 |
| cg18028711 | 0.298 | TRUE | 0.359 | 0.356 |
| cg02011074 | 0.298 | TRUE | 0.257 | 0.335 |
| cg09059319 | 0.297 | TRUE | 0.255 | 0.256 |
| cg01351041 | 0.297 | TRUE | 0.201 | 0.270 |
| cg17384889 | 0.297 | TRUE | 0.298 | 0.252 |
| cg04518342 | 0.297 | TRUE | 0.291 | 0.351 |
| cg15655500 | 0.297 | TRUE | 0.225 | 0.273 |
| cg18449964 | 0.297 | TRUE | 0.249 | 0.281 |
| cg08869573 | 0.297 | TRUE | 0.252 | 0.256 |
| cg16848524 | 0.297 | TRUE | 0.414 | 0.473 |
| cg07207982 | 0.297 | TRUE | 0.353 | 0.364 |
| cg22378919 | 0.297 | TRUE | 0.260 | 0.228 |
| cg24505618 | 0.297 | TRUE | 0.324 | 0.327 |
| cg07274716 | 0.297 | TRUE | 0.420 | 0.385 |
| cg27071460 | 0.297 | TRUE | 0.295 | 0.309 |
| cg03671191 | 0.297 | TRUE | 0.311 | 0.369 |
| cg01373292 | 0.297 | TRUE | 0.199 | 0.325 |
| cg25537993 | 0.297 | TRUE | 0.479 | 0.467 |
| cg23045908 | 0.297 | TRUE | 0.173 | 0.171 |
| cg07314384 | 0.297 | NA | NA | NA |
| cg13950829 | 0.297 | NA | NA | 0.359 |
| cg21357629 | 0.296 | NA | NA | 0.424 |
| cg22160000 | 0.296 | TRUE | 0.258 | 0.264 |
| cg14414971 | 0.296 | TRUE | 0.262 | 0.375 |
| cg13847070 | 0.296 | TRUE | 0.242 | 0.182 |
| cg00407600 | 0.296 | NA | NA | NA |
| cg23200020 | 0.296 | TRUE | 0.334 | 0.331 |
| cg09908110 | 0.296 | TRUE | 0.186 | 0.266 |
| cg07836661 | 0.296 | NA | NA | 0.276 |
| cg16779463 | 0.296 | TRUE | 0.309 | 0.424 |
| cg06014958 | 0.296 | TRUE | 0.285 | 0.287 |
| cg08049853 | 0.296 | TRUE | 0.288 | 0.293 |
| cg07671858 | 0.296 | TRUE | 0.298 | 0.244 |
| cg19385331 | 0.296 | TRUE | 0.239 | 0.231 |
| cg18935453 | 0.296 | TRUE | 0.279 | 0.289 |
| cg10723962 | 0.296 | TRUE | 0.266 | 0.233 |
| cg20743280 | 0.296 | TRUE | 0.291 | 0.266 |
| cg01480180 | 0.296 | TRUE | 0.318 | 0.292 |
| cg01857475 | 0.296 | NA | NA | 0.332 |
| cg16871763 | 0.296 | TRUE | 0.190 | 0.209 |
| cg05057910 | 0.295 | TRUE | 0.275 | 0.261 |
| cg05036656 | 0.295 | TRUE | 0.305 | 0.273 |
| cg08901901 | 0.295 | TRUE | 0.282 | 0.280 |
| cg25331703 | 0.295 | TRUE | 0.267 | 0.348 |
| cg09240763 | 0.295 | TRUE | 0.291 | 0.288 |
| cg26509691 | 0.295 | TRUE | 0.307 | 0.270 |
| cg23244790 | 0.295 | TRUE | 0.378 | 0.376 |
| cg08769844 | 0.295 | NA | NA | NA |
| cg16194233 | 0.295 | TRUE | 0.217 | 0.235 |
| cg02823783 | 0.295 | TRUE | 0.294 | 0.391 |
| cg12248614 | 0.295 | TRUE | 0.365 | 0.390 |
| cg03217795 | 0.295 | TRUE | 0.328 | 0.383 |
| cg09614653 | 0.295 | TRUE | 0.184 | 0.225 |
| cg03655683 | 0.295 | TRUE | 0.325 | 0.319 |
| cg14871601 | 0.295 | TRUE | 0.240 | 0.250 |
| cg22794031 | 0.295 | NA | NA | NA |
| cg12534150 | 0.295 | TRUE | 0.248 | 0.341 |
| cg20685897 | 0.295 | TRUE | 0.229 | 0.261 |
| cg16562275 | 0.295 | TRUE | 0.269 | 0.255 |
| cg01227537 | 0.295 | NA | NA | 0.401 |
| cg08189989 | 0.294 | NA | NA | 0.311 |
| cg00866399 | 0.294 | TRUE | 0.213 | 0.198 |
| cg12457909 | 0.294 | TRUE | 0.281 | 0.273 |
| cg23322933 | 0.294 | TRUE | 0.397 | 0.394 |
| cg19587420 | 0.294 | NA | NA | NA |
| cg27069372 | 0.294 | NA | NA | NA |
| cg15535138 | 0.294 | NA | NA | NA |
| cg12855166 | 0.294 | TRUE | 0.247 | 0.301 |
| cg23229261 | 0.294 | TRUE | 0.426 | 0.381 |
| cg09162333 | 0.294 | NA | NA | NA |
| cg13891181 | 0.294 | TRUE | 0.249 | 0.260 |
| cg09574088 | 0.294 | TRUE | 0.277 | 0.352 |
| cg06816106 | 0.294 | TRUE | 0.196 | 0.275 |
| cg23305408 | 0.294 | TRUE | 0.191 | 0.185 |
| cg04806107 | 0.294 | NA | NA | NA |
| cg06635799 | 0.294 | NA | NA | NA |
| cg23881278 | 0.294 | TRUE | 0.372 | 0.364 |
| cg06673178 | 0.294 | TRUE | 0.227 | 0.240 |
| cg00347904 | 0.294 | TRUE | 0.391 | 0.291 |
| cg09461837 | 0.294 | TRUE | 0.288 | 0.344 |
| cg27249419 | 0.294 | NA | NA | 0.304 |
| cg14486338 | 0.293 | TRUE | 0.492 | 0.402 |
| cg27262412 | 0.293 | TRUE | 0.265 | 0.301 |
| cg23874561 | 0.293 | TRUE | 0.244 | 0.317 |
| cg06686029 | 0.293 | TRUE | 0.320 | 0.360 |
| cg19554255 | 0.293 | TRUE | 0.262 | 0.331 |
| cg16909733 | 0.293 | TRUE | 0.286 | 0.271 |
| cg24721899 | 0.293 | TRUE | 0.341 | 0.376 |
| cg11261698 | 0.293 | NA | NA | NA |
| cg02781618 | 0.293 | TRUE | 0.263 | 0.258 |
| cg19763461 | 0.293 | NA | NA | 0.271 |
| cg03699623 | 0.293 | TRUE | 0.206 | 0.302 |
| cg19779211 | 0.293 | TRUE | 0.271 | 0.306 |
| cg21489390 | 0.293 | NA | NA | NA |
| cg05184938 | 0.293 | TRUE | 0.248 | 0.291 |
| cg03896611 | 0.293 | TRUE | 0.215 | 0.236 |
| cg27221797 | 0.293 | NA | NA | NA |
| cg21392341 | 0.293 | TRUE | 0.337 | 0.350 |
| cg11068343 | 0.293 | TRUE | 0.220 | 0.298 |
| cg22830113 | 0.293 | TRUE | 0.411 | 0.437 |
| cg00705992 | 0.293 | TRUE | 0.290 | 0.302 |
| cg16788319 | 0.292 | NA | NA | NA |
| cg14247643 | 0.292 | NA | NA | NA |
| cg13577628 | 0.292 | NA | NA | 0.215 |
| cg08042316 | 0.292 | TRUE | 0.296 | 0.356 |
| cg02668581 | 0.292 | TRUE | 0.109 | 0.194 |
| cg09060057 | 0.292 | NA | NA | NA |
| cg27363327 | 0.292 | NA | NA | 0.352 |
| cg12356890 | 0.292 | TRUE | 0.361 | 0.384 |
| cg12749863 | 0.292 | TRUE | 0.317 | 0.339 |
| cg11001844 | 0.292 | NA | NA | NA |
| cg13736514 | 0.292 | TRUE | 0.233 | 0.247 |
| cg20056542 | 0.292 | TRUE | 0.323 | 0.357 |
| cg02620769 | 0.292 | TRUE | 0.170 | 0.192 |
| cg06100807 | 0.292 | TRUE | 0.277 | 0.282 |
| cg08624507 | 0.292 | NA | NA | NA |
| cg03850256 | 0.292 | TRUE | 0.298 | 0.214 |
| cg03673965 | 0.292 | TRUE | 0.278 | 0.279 |

**Table S3. List of 132 hypermethylation markers with *p* < 0.05, Δβ > 0.3 and β_normal_ < 0.1.**

| Target ID | P.Value | Adjust. Pval. | Beta. Diff. | Mean  HCC | Mean  Normal | UCSC RefGene  Name | Chr. | MapInfo | UCSC CpG  Islands Name | Methyl450  Loci |
| --- | --- | --- | --- | --- | --- | --- | --- | --- | --- | --- |
| cg20172627 | 7.78E-28 | 3.58E-24 | 0.463 | 0.550 | 0.088 |  | 2 | 25439110 | chr2:25438725-25439276 | TRUE |
| cg12701184 | 8.91E-24 | 8.14E-21 | 0.423 | 0.509 | 0.086 | USP44 | 12 | 95942212 | chr12:95941906-95942979 | NA |
| cg20288165 | 8.99E-23 | 5.37E-20 | 0.405 | 0.477 | 0.072 | LOXL3;DOK1 | 2 | 74782157 | chr2:74781494-74782685 | NA |
| cg14570307 | 3.21E-25 | 5.18E-22 | 0.402 | 0.497 | 0.096 | ZNF783 | 7 | 148982284 | chr7:148982079-148982675 | TRUE |
| cg04823311 | 1.34E-23 | 1.13E-20 | 0.394 | 0.487 | 0.094 | TRIL | 7 | 28997485 | chr7:28995305-28998541 | TRUE |
| cg11595545 | 5.34E-21 | 1.53E-18 | 0.394 | 0.481 | 0.087 | KCNA3 | 1 | 111217497 | chr1:111216244-111217937 | TRUE |
| cg14263942 | 1.24E-22 | 6.96E-20 | 0.387 | 0.487 | 0.100 | CDKL2 | 4 | 76555772 | chr4:76555366-76556079 | TRUE |
| cg07689503 | 4.79E-21 | 1.40E-18 | 0.386 | 0.475 | 0.089 | MTHFD2 | 2 | 74426200 | chr2:74425444-74426423 | TRUE |
| cg15457058 | 2.29E-21 | 7.62E-19 | 0.383 | 0.469 | 0.087 | FOXE3 | 1 | 47882322 | chr1:47881896-47883065 | TRUE |
| cg12840719 | 8.71E-20 | 1.51E-17 | 0.382 | 0.460 | 0.077 | CDKN2A | 9 | 21968233 | chr9:21968358-21968728 | TRUE |
| cg25214789 | 3.20E-24 | 3.50E-21 | 0.381 | 0.477 | 0.096 | C5orf49 | 5 | 7850070 | chr5:7849945-7850439 | TRUE |
| cg10376598 | 1.57E-18 | 1.61E-16 | 0.379 | 0.423 | 0.044 | LPAR2 | 19 | 19739411 | chr19:19738572-19739821 | TRUE |
| cg17300544 | 7.51E-15 | 1.84E-13 | 0.377 | 0.454 | 0.077 | SEPT9 | 17 | 75369091 | chr17:75368688-75370506 | TRUE |
| cg21790626 | 7.76E-23 | 4.79E-20 | 0.376 | 0.458 | 0.082 | ZNF154 | 19 | 58220494 | chr19:58220189-58220517 | TRUE |
| cg03918605 | 3.46E-22 | 1.60E-19 | 0.376 | 0.436 | 0.060 | USP44 | 12 | 95942081 | chr12:95941906-95942979 | NA |
| cg00256076 | 1.79E-22 | 9.34E-20 | 0.374 | 0.451 | 0.077 | USP44 | 12 | 95942086 | chr12:95941906-95942979 | NA |
| cg17154724 | 1.33E-18 | 1.40E-16 | 0.373 | 0.471 | 0.099 | DNM3 | 1 | 171810322 | chr1:171810467-171811325 | TRUE |
| cg00922376 | 2.52E-17 | 1.55E-15 | 0.372 | 0.461 | 0.089 | PRDM2 | 1 | 14026584 | chr1:14026481-14027200 | TRUE |
| cg23982858 | 1.31E-20 | 3.17E-18 | 0.372 | 0.455 | 0.083 | USP44 | 12 | 95941869 | chr12:95941906-95942979 | TRUE |
| cg16579555 | 3.22E-24 | 3.51E-21 | 0.371 | 0.448 | 0.076 | RNF135 | 17 | 29298352 | chr17:29298046-29298606 | TRUE |
| cg04845566 | 1.07E-26 | 3.09E-23 | 0.368 | 0.450 | 0.082 | ZNF783;LOC155060 | 7 | 148982278 | chr7:148982079-148982675 | NA |
| cg11672054 | 5.54E-20 | 1.04E-17 | 0.365 | 0.418 | 0.053 | HIST3H2BB;HIST3H2A | 1 | 228645482 | chr1:228645196-228646434 | TRUE |
| cg14988503 | 3.68E-22 | 1.68E-19 | 0.360 | 0.414 | 0.054 | CDKL2 | 4 | 76555547 | chr4:76555366-76556079 | TRUE |
| cg02659794 | 1.12E-18 | 1.22E-16 | 0.359 | 0.427 | 0.069 | LDHB | 12 | 21810750 | chr12:21810488-21810766 | TRUE |
| cg25767001 | 5.11E-21 | 1.48E-18 | 0.358 | 0.410 | 0.052 | USP44 | 12 | 95942100 | chr12:95941906-95942979 | NA |
| cg18555909 | 8.44E-16 | 2.89E-14 | 0.356 | 0.449 | 0.093 | PRDM2 | 1 | 14026587 | chr1:14026481-14027200 | NA |
| cg02864844 | 3.35E-17 | 1.96E-15 | 0.356 | 0.435 | 0.079 |  | 7 | 149917263 | chr7:149916640-149918264 | TRUE |
| cg00948275 | 1.83E-18 | 1.82E-16 | 0.355 | 0.436 | 0.081 | HIST3H2BB;HIST3H2A | 1 | 228645512 | chr1:228645196-228646434 | TRUE |
| cg20971220 | 2.83E-19 | 3.96E-17 | 0.355 | 0.396 | 0.042 | FLJ26850 | 19 | 50554067 | chr19:50554012-50554492 | TRUE |
| cg10648670 | 1.16E-17 | 8.20E-16 | 0.353 | 0.442 | 0.088 |  | 2 | 25439375 | chr2:25438725-25439276 | TRUE |
| cg17297071 | 1.93E-18 | 1.90E-16 | 0.353 | 0.417 | 0.064 | MIR155HG | 21 | 26934424 | chr21:26934423-26934805 | TRUE |
| cg08571241 | 4.31E-20 | 8.45E-18 | 0.352 | 0.418 | 0.066 | USP44 | 12 | 95942122 | chr12:95941906-95942979 | NA |
| cg19306047 | 1.68E-16 | 7.48E-15 | 0.351 | 0.408 | 0.057 | LPAR2 | 19 | 19739407 | chr19:19738572-19739821 | TRUE |
| cg03621881 | 1.16E-14 | 2.67E-13 | 0.351 | 0.448 | 0.097 | BRUNOL6 | 15 | 72612817 | chr15:72611946-72612802 | TRUE |
| cg26005578 | 7.88E-19 | 9.15E-17 | 0.347 | 0.402 | 0.055 | RASSF10 | 11 | 13030676 | chr11:13030330-13032584 | TRUE |
| cg23433889 | 2.93E-20 | 6.14E-18 | 0.347 | 0.383 | 0.035 | MIR155HG | 21 | 26934455 | chr21:26934423-26934805 | TRUE |
| cg00573330 | 2.21E-17 | 1.40E-15 | 0.347 | 0.415 | 0.068 | ZNF141 | 4 | 330651 | chr4:330162-332068 | TRUE |
| cg21176475 | 3.31E-15 | 9.16E-14 | 0.347 | 0.440 | 0.094 | LRRC43 | 12 | 122667627 | chr12:122667648-122668038 | TRUE |
| cg20275528 | 4.95E-18 | 4.10E-16 | 0.346 | 0.422 | 0.076 | SEPT9 | 17 | 75369484 | chr17:75368688-75370506 | TRUE |
| cg01204606 | 5.73E-18 | 4.60E-16 | 0.344 | 0.441 | 0.097 |  | 11 | 116451788 | chr11:116450659-116451912 | TRUE |
| cg24393316 | 7.30E-17 | 3.74E-15 | 0.343 | 0.442 | 0.098 | FOXE1 | 9 | 100616469 | chr9:100615234-100617510 | TRUE |
| cg26989048 | 1.51E-17 | 1.01E-15 | 0.343 | 0.399 | 0.057 | C8orf88 | 8 | 91997672 | chr8:91997230-91997906 | NA |
| cg18202336 | 1.47E-16 | 6.70E-15 | 0.342 | 0.400 | 0.058 | IGF1R | 15 | 99193993 | chr15:99190446-99194559 | TRUE |
| cg13185030 | 1.96E-11 | 1.86E-10 | 0.341 | 0.428 | 0.087 | LGALS3 | 14 | 55595949 | chr14:55595697-55596692 | TRUE |
| cg03308628 | 4.48E-19 | 5.80E-17 | 0.341 | 0.413 | 0.072 | USP44 | 12 | 95942287 | chr12:95941906-95942979 | TRUE |
| cg11957331 | 4.47E-17 | 2.49E-15 | 0.341 | 0.434 | 0.092 | TMEM145 | 19 | 42828156 | chr19:42827743-42829149 | TRUE |
| cg22806907 | 8.50E-17 | 4.24E-15 | 0.340 | 0.409 | 0.068 | PLEKHH3 | 17 | 40826212 | chr17:40827975-40828659 | TRUE |
| cg23337116 | 2.17E-20 | 4.78E-18 | 0.339 | 0.413 | 0.073 | RASL11B | 4 | 53728510 | chr4:53728037-53729000 | TRUE |
| cg14209005 | 3.29E-19 | 4.49E-17 | 0.339 | 0.421 | 0.082 | LDHB | 12 | 21810852 | chr12:21810488-21810766 | TRUE |
| cg22789900 | 2.92E-17 | 1.75E-15 | 0.337 | 0.416 | 0.079 | MIXL1 | 1 | 226411715 | chr1:226411007-226411880 | TRUE |
| cg18153401 | 2.28E-13 | 3.54E-12 | 0.336 | 0.369 | 0.033 | CDKN2A | 9 | 21974871 | chr9:21974578-21975306 | NA |
| cg02161084 | 1.62E-20 | 3.77E-18 | 0.334 | 0.379 | 0.044 | CD276 | 15 | 73989526 |  | TRUE |
| cg26117023 | 3.93E-18 | 3.38E-16 | 0.334 | 0.376 | 0.042 | LOXL3;DOK1 | 2 | 74782096 | chr2:74781494-74782685 | TRUE |
| cg19729116 | 4.92E-14 | 9.33E-13 | 0.333 | 0.415 | 0.082 | BTN1A1 | 6 | 26501981 | chr6:26501725-26502107 | TRUE |
| cg02373152 | 3.22E-14 | 6.46E-13 | 0.332 | 0.432 | 0.100 | PAQR8 | 6 | 52227767 | chr6:52226753-52228006 | TRUE |
| cg06180621 | 2.13E-17 | 1.35E-15 | 0.332 | 0.381 | 0.049 | DNAH10 | 12 | 124246917 | chr12:124246524-124247254 | NA |
| cg12177220 | 3.92E-22 | 1.77E-19 | 0.332 | 0.409 | 0.077 | DLEC1 | 3 | 38080925 | chr3:38080628-38081187 | TRUE |
| cg00527440 | 3.63E-13 | 5.33E-12 | 0.332 | 0.413 | 0.081 | THAP4 | 2 | 242549864 | chr2:242549373-242549995 | TRUE |
| cg01563704 | 2.15E-16 | 9.17E-15 | 0.331 | 0.399 | 0.068 | HIST1H3G;HIST1H2BI | 6 | 26271816 | chr6:26272470-26272696 | TRUE |
| cg20401551 | 2.86E-13 | 4.32E-12 | 0.331 | 0.387 | 0.056 | SCARF2 | 22 | 20790985 | chr22:20790638-20792665 | TRUE |
| cg01362570 | 5.39E-15 | 1.38E-13 | 0.330 | 0.387 | 0.057 | SLC25A36 | 3 | 140660572 | chr3:140660334-140661602 | TRUE |
| cg22497741 | 4.03E-20 | 7.99E-18 | 0.329 | 0.396 | 0.067 | C1orf70 | 1 | 1475644 | chr1:1476093-1476669 | TRUE |
| cg24724633 | 8.26E-15 | 2.00E-13 | 0.329 | 0.390 | 0.062 | ZFP82 | 19 | 36909413 | chr19:36909281-36909854 | TRUE |
| cg14032025 | 9.89E-25 | 1.34E-21 | 0.328 | 0.417 | 0.089 | ZFP64 | 20 | 50721129 | chr20:50721310-50721912 | TRUE |
| cg08584377 | 5.68E-14 | 1.06E-12 | 0.326 | 0.411 | 0.086 | FSCN1 | 7 | 5632269 | chr7:5632335-5634555 | TRUE |
| cg11411872 | 1.74E-20 | 4.01E-18 | 0.325 | 0.401 | 0.075 | C1QL3 | 10 | 16563096 | chr10:16561604-16563822 | NA |
| cg04907523 | 4.59E-13 | 6.55E-12 | 0.325 | 0.390 | 0.064 | VASH2 | 1 | 213124896 | chr1:213123647-213125092 | TRUE |
| cg10716835 | 7.29E-17 | 3.73E-15 | 0.324 | 0.396 | 0.072 | CYP26A1 | 10 | 94834582 | chr10:94833272-94835256 | TRUE |
| cg18456523 | 1.05E-12 | 1.37E-11 | 0.323 | 0.422 | 0.098 |  | 5 | 54516805 | chr5:54516267-54516919 | TRUE |
| cg02038168 | 3.22E-15 | 8.96E-14 | 0.323 | 0.411 | 0.088 |  | 22 | 39784481 | chr22:39784354-39785104 | TRUE |
| cg24957851 | 5.05E-14 | 9.54E-13 | 0.323 | 0.369 | 0.046 |  | X | 39868397 | chrX:39864525-39869106 | NA |
| cg21416498 | 3.41E-18 | 3.01E-16 | 0.323 | 0.357 | 0.034 | AMN | 14 | 103395021 | chr14:103394397-103397070 | NA |
| cg24425021 | 1.30E-19 | 2.09E-17 | 0.323 | 0.407 | 0.085 | POU4F1 | 13 | 79177520 | chr13:79175610-79177985 | TRUE |
| cg18794404 | 3.07E-20 | 6.40E-18 | 0.323 | 0.401 | 0.079 |  | 10 | 22542024 | chr10:22540707-22542739 | TRUE |
| cg06893138 | 8.61E-18 | 6.42E-16 | 0.322 | 0.369 | 0.047 | HIST3H2BB;HIST3H2A | 1 | 228645306 | chr1:228645196-228646434 | TRUE |
| cg08100565 | 1.48E-16 | 6.72E-15 | 0.322 | 0.385 | 0.063 | SLC25A36 | 3 | 140660748 | chr3:140660334-140661602 | TRUE |
| cg18210732 | 3.67E-14 | 7.24E-13 | 0.322 | 0.406 | 0.084 | FGF19 | 11 | 69518545 | chr11:69517840-69519929 | TRUE |
| cg26468478 | 2.89E-17 | 1.74E-15 | 0.322 | 0.388 | 0.066 | CELSR3 | 3 | 48693597 | chr3:48693118-48694768 | TRUE |
| cg01447112 | 1.48E-20 | 3.51E-18 | 0.321 | 0.417 | 0.096 |  | 7 | 6703803 | chr7:6703503-6704075 | TRUE |
| cg06848185 | 2.96E-17 | 1.77E-15 | 0.321 | 0.399 | 0.078 | SEPT9 | 17 | 75368902 | chr17:75368688-75370506 | TRUE |
| cg21027189 | 6.25E-19 | 7.55E-17 | 0.319 | 0.373 | 0.054 | ZFP64 | 20 | 50721658 | chr20:50721310-50721912 | NA |
| cg07042902 | 3.47E-13 | 5.11E-12 | 0.319 | 0.381 | 0.062 | CDKN2A | 9 | 21974943 | chr9:21974578-21975306 | NA |
| cg26992213 | 3.65E-13 | 5.35E-12 | 0.316 | 0.372 | 0.056 |  | 6 | 159290838 | chr6:159290673-159291195 | TRUE |
| cg01162037 | 5.78E-16 | 2.09E-14 | 0.316 | 0.384 | 0.068 | TTYH1 | 19 | 54926623 | chr19:54927902-54928225 | NA |
| cg10786045 | 3.15E-14 | 6.33E-13 | 0.316 | 0.373 | 0.057 | CELF6 | 15 | 72612466 | chr15:72611946-72612802 | NA |
| cg19497031 | 9.25E-20 | 1.58E-17 | 0.316 | 0.397 | 0.081 | POU4F1 | 13 | 79176272 | chr13:79175610-79177985 | TRUE |
| cg21453443 | 4.34E-16 | 1.65E-14 | 0.315 | 0.399 | 0.084 | HIST3H2A;HIST3H2BB | 1 | 228645627 | chr1:228645196-228646434 | TRUE |
| cg17716617 | 2.24E-15 | 6.58E-14 | 0.315 | 0.397 | 0.082 | KIAA1522 | 1 | 33219580 | chr1:33219427-33220028 | TRUE |
| cg12874092 | 3.07E-16 | 1.23E-14 | 0.315 | 0.353 | 0.038 | VIM | 10 | 17271519 | chr10:17270430-17272617 | TRUE |
| cg24427504 | 1.47E-21 | 5.28E-19 | 0.314 | 0.402 | 0.088 |  | 12 | 133481520 | chr12:133484658-133485739 | TRUE |
| cg06188229 | 8.83E-17 | 4.38E-15 | 0.313 | 0.412 | 0.099 | MIXL1 | 1 | 226411818 | chr1:226411007-226411880 | TRUE |
| cg24091001 | 2.59E-17 | 1.58E-15 | 0.312 | 0.410 | 0.098 | DLX1 | 2 | 172949870 | chr2:172949242-172950126 | NA |
| cg03804136 | 5.28E-14 | 9.93E-13 | 0.312 | 0.384 | 0.072 | SEPT9 | 17 | 75369219 | chr17:75368688-75370506 | TRUE |
| cg06377278 | 1.78E-13 | 2.85E-12 | 0.311 | 0.385 | 0.074 | RUNX3 | 1 | 25256369 | chr1:25255527-25259005 | TRUE |
| cg02728595 | 8.50E-16 | 2.90E-14 | 0.311 | 0.407 | 0.096 |  | 3 | 196255632 | chr3:196255495-196256013 | TRUE |
| cg00778995 | 1.08E-13 | 1.84E-12 | 0.311 | 0.382 | 0.071 | POU3F3 | 2 | 105470558 | chr2:105468851-105473488 | TRUE |
| cg20803547 | 6.63E-17 | 3.45E-15 | 0.311 | 0.365 | 0.054 | IL12RB2 | 1 | 67773440 | chr1:67773329-67773767 | TRUE |
| cg19741167 | 2.41E-17 | 1.49E-15 | 0.311 | 0.393 | 0.082 | POLR1A | 2 | 86263270 |  | TRUE |
| cg14250130 | 2.55E-15 | 7.35E-14 | 0.311 | 0.386 | 0.075 | PFKP | 10 | 3109361 | chr10:3108650-3111419 | TRUE |
| cg18818834 | 4.85E-13 | 6.88E-12 | 0.310 | 0.380 | 0.070 | PAQR8 | 6 | 52226764 | chr6:52226753-52228006 | TRUE |
| cg18601167 | 2.87E-15 | 8.12E-14 | 0.309 | 0.396 | 0.086 | PRKAR1B | 7 | 752286 | chr7:750788-751237 | TRUE |
| cg02433564 | 5.44E-16 | 1.99E-14 | 0.309 | 0.379 | 0.070 |  | 6 | 37673335 | chr6:37673308-37673774 | TRUE |
| cg00810208 | 7.60E-16 | 2.64E-14 | 0.309 | 0.395 | 0.086 | IDUA | 4 | 997012 | chr4:995482-997541 | TRUE |
| cg05040544 | 6.59E-13 | 9.02E-12 | 0.309 | 0.373 | 0.065 | EFNB2 | 13 | 107188256 | chr13:107186468-107189024 | TRUE |
| cg15467646 | 8.95E-22 | 3.52E-19 | 0.308 | 0.373 | 0.065 | LDLRAD2 | 1 | 22141014 | chr1:22140891-22141407 | TRUE |
| cg15044248 | 5.78E-14 | 1.07E-12 | 0.308 | 0.385 | 0.077 | SEPT9 | 17 | 75369224 | chr17:75368688-75370506 | TRUE |
| cg25884711 | 4.20E-15 | 1.12E-13 | 0.308 | 0.365 | 0.057 | NPY | 7 | 24323840 | chr7:24323558-24325080 | TRUE |
| cg16489586 | 5.27E-15 | 1.36E-13 | 0.308 | 0.406 | 0.098 | ATL1 | 14 | 51027964 | chr14:51026872-51027570 | TRUE |
| cg27212234 | 6.57E-16 | 2.33E-14 | 0.308 | 0.366 | 0.058 | ATP4A | 19 | 36048757 | chr19:36048556-36049673 | TRUE |
| cg17306261 | 1.75E-15 | 5.34E-14 | 0.307 | 0.403 | 0.096 | C14orf50 | 14 | 65016545 | chr14:65016516-65016909 | TRUE |
| cg21872764 | 4.87E-20 | 9.32E-18 | 0.307 | 0.402 | 0.094 | CLDN5 | 22 | 19510977 | chr22:19510871-19512254 | TRUE |
| cg18016826 | 1.29E-15 | 4.12E-14 | 0.307 | 0.401 | 0.094 | NSD1 | 5 | 176560001 | chr5:176558852-176561652 | TRUE |
| cg07844931 | 3.38E-15 | 9.34E-14 | 0.307 | 0.359 | 0.052 | TEPP | 16 | 58018880 | chr16:58018400-58019471 | TRUE |
| cg17813946 | 1.21E-12 | 1.55E-11 | 0.306 | 0.349 | 0.043 |  | 22 | 39784464 | chr22:39784354-39785104 | NA |
| cg05115157 | 5.23E-19 | 6.54E-17 | 0.306 | 0.368 | 0.062 | LRRN1 | 3 | 3840655 | chr3:3840513-3842772 | NA |
| cg21875802 | 1.23E-16 | 5.76E-15 | 0.305 | 0.401 | 0.096 |  | 2 | 45231382 | chr2:45231211-45231482 | TRUE |
| cg10896862 | 5.10E-18 | 4.19E-16 | 0.305 | 0.385 | 0.080 | MPZ | 1 | 161275561 |  | TRUE |
| cg05578357 | 2.50E-15 | 7.24E-14 | 0.305 | 0.392 | 0.087 | LDHB | 12 | 21810868 | chr12:21810488-21810766 | TRUE |
| cg20093476 | 1.47E-15 | 4.62E-14 | 0.305 | 0.397 | 0.092 | SPDYA | 2 | 29033286 | chr2:29033351-29034011 | NA |
| cg14024461 | 7.64E-22 | 3.07E-19 | 0.305 | 0.386 | 0.081 | HIST3H2A;HIST3H2BB | 1 | 228646047 | chr1:228645196-228646434 | TRUE |
| cg21347053 | 1.01E-23 | 9.03E-21 | 0.304 | 0.390 | 0.086 | GMDS | 6 | 1624978 | chr6:1624185-1625468 | TRUE |
| cg12450224 | 2.41E-12 | 2.85E-11 | 0.303 | 0.338 | 0.035 | LOC101928386 | 2 | 144694581 | chr2:144694666-144695180 | NA |
| cg26983469 | 1.44E-16 | 6.57E-15 | 0.303 | 0.367 | 0.063 | VIM | 10 | 17271051 | chr10:17270430-17272617 | TRUE |
| cg01725199 | 2.10E-15 | 6.24E-14 | 0.303 | 0.359 | 0.056 | GALNT12 | 9 | 101569990 | chr9:101569905-101570349 | TRUE |
| cg16915821 | 3.29E-18 | 2.92E-16 | 0.303 | 0.374 | 0.071 | DKK3 | 11 | 12030187 | chr11:12029737-12030841 | TRUE |
| cg21461649 | 1.64E-13 | 2.66E-12 | 0.303 | 0.402 | 0.100 | CFTR | 7 | 117119938 |  | TRUE |
| cg08296831 | 5.87E-14 | 1.09E-12 | 0.302 | 0.400 | 0.097 | OVOL1 | 11 | 65554175 | chr11:65553749-65555573 | TRUE |
| cg10900437 | 1.37E-16 | 6.30E-15 | 0.302 | 0.358 | 0.056 | UST | 6 | 149068864 | chr6:149068179-149069775 | TRUE |
| cg17975443 | 4.84E-15 | 1.26E-13 | 0.301 | 0.390 | 0.089 | TBX4 | 17 | 59534998 | chr17:59531723-59535254 | TRUE |
| cg15845792 | 1.23E-19 | 2.00E-17 | 0.300 | 0.387 | 0.086 |  | 6 | 28175446 | chr6:28175282-28175605 | TRUE |
| cg18233405 | 3.55E-26 | 8.46E-23 | 0.300 | 0.399 | 0.099 | TSPYL5 | 8 | 98290148 | chr8:98289604-98290404 | TRUE |
| cg21908638 | 4.83E-16 | 1.80E-14 | 0.300 | 0.390 | 0.090 | FAM55C | 3 | 101497982 | chr3:101497830-101498648 | TRUE |

**Table S4. List of 32 markers further screened from Table S3, with AUC > 0.85 and Youden Index (YI) ≥ 0.8.**

| Target ID | Beta. Diff. | Mean  HCC | Mean  Normal | AUC | Cutoff | Sensitivity | Specificity | Youden  Index | UCSC RefGene  Name |
| --- | --- | --- | --- | --- | --- | --- | --- | --- | --- |
| cg20172627 | 0.463 | 0.550 | 0.088 | 0.900 | 0.224 | 0.883 | 0.983 | 0.866 |  |
| cg12701184 | 0.423 | 0.509 | 0.086 | 0.898 | 0.218 | 0.833 | 1.000 | 0.833 | USP44 |
| cg20288165 | 0.405 | 0.477 | 0.072 | 0.872 | 0.206 | 0.817 | 1.000 | 0.817 | LOXL3;DOK1 |
| cg14570307 | 0.402 | 0.497 | 0.096 | 0.914 | 0.242 | 0.833 | 1.000 | 0.833 | ZNF783;LOC155060 |
| cg04823311 | 0.394 | 0.487 | 0.094 | 0.886 | 0.167 | 0.833 | 1.000 | 0.833 | TRIL |
| cg14263942 | 0.387 | 0.487 | 0.100 | 0.879 | 0.260 | 0.833 | 1.000 | 0.833 | CDKL2 |
| cg07689503 | 0.386 | 0.475 | 0.089 | 0.872 | 0.172 | 0.833 | 0.983 | 0.816 | MTHFD2 |
| cg15457058 | 0.383 | 0.469 | 0.087 | 0.867 | 0.179 | 0.817 | 1.000 | 0.817 | FOXE3 |
| cg12840719 | 0.382 | 0.460 | 0.077 | 0.917 | 0.150 | 0.800 | 1.000 | 0.800 | CDKN2A |
| cg25214789 | 0.381 | 0.477 | 0.096 | 0.913 | 0.185 | 0.833 | 0.983 | 0.816 | C5orf49 |
| cg21790626 | 0.376 | 0.458 | 0.082 | 0.901 | 0.149 | 0.867 | 0.967 | 0.834 | ZNF154 |
| cg03918605 | 0.376 | 0.436 | 0.060 | 0.883 | 0.202 | 0.800 | 1.000 | 0.800 | USP44 |
| cg00256076 | 0.374 | 0.451 | 0.077 | 0.873 | 0.164 | 0.833 | 0.967 | 0.800 | USP44 |
| cg23982858 | 0.372 | 0.455 | 0.083 | 0.891 | 0.154 | 0.817 | 1.000 | 0.817 | USP44 |
| cg16579555 | 0.371 | 0.448 | 0.076 | 0.870 | 0.258 | 0.833 | 1.000 | 0.833 | RNF135 |
| cg04845566 | 0.368 | 0.450 | 0.082 | 0.927 | 0.154 | 0.867 | 0.983 | 0.850 | ZNF783;LOC155060 |
| cg14988503 | 0.360 | 0.414 | 0.054 | 0.915 | 0.096 | 0.867 | 0.967 | 0.834 | CDKL2 |
| cg25767001 | 0.358 | 0.410 | 0.052 | 0.884 | 0.120 | 0.833 | 0.983 | 0.816 | USP44 |
| cg20971220 | 0.355 | 0.396 | 0.042 | 0.880 | 0.073 | 0.833 | 0.983 | 0.816 | FLJ26850 |
| cg26005578 | 0.347 | 0.402 | 0.055 | 0.919 | 0.107 | 0.800 | 1.000 | 0.800 | RASSF10 |
| cg23433889 | 0.347 | 0.383 | 0.035 | 0.893 | 0.087 | 0.817 | 1.000 | 0.817 | MIR155HG |
| cg03308628 | 0.341 | 0.413 | 0.072 | 0.896 | 0.141 | 0.817 | 0.983 | 0.800 | USP44 |
| cg02161084 | 0.334 | 0.379 | 0.044 | 0.897 | 0.085 | 0.800 | 1.000 | 0.800 | CD276 |
| cg12177220 | 0.332 | 0.409 | 0.077 | 0.899 | 0.159 | 0.850 | 0.967 | 0.817 | DLEC1 |
| cg14032025 | 0.328 | 0.417 | 0.089 | 0.921 | 0.159 | 0.833 | 0.983 | 0.816 | ZFP64 |
| cg01447112 | 0.321 | 0.417 | 0.096 | 0.901 | 0.131 | 0.867 | 0.933 | 0.800 |  |
| cg19497031 | 0.316 | 0.397 | 0.081 | 0.879 | 0.141 | 0.800 | 1.000 | 0.800 | POU4F1 |
| cg24427504 | 0.314 | 0.402 | 0.088 | 0.879 | 0.132 | 0.817 | 1.000 | 0.817 |  |
| cg15467646 | 0.308 | 0.373 | 0.065 | 0.886 | 0.125 | 0.833 | 1.000 | 0.833 | LDLRAD2 |
| cg21347053 | 0.304 | 0.390 | 0.086 | 0.899 | 0.167 | 0.850 | 0.967 | 0.817 | GMDS |
| cg15845792 | 0.300 | 0.387 | 0.086 | 0.925 | 0.174 | 0.800 | 1.000 | 0.800 |  |
| cg18233405 | 0.300 | 0.399 | 0.099 | 0.933 | 0.240 | 0.850 | 1.000 | 0.850 | TSPYL5 |

**Table S5. Characteristics of study participants in plasma pilot study.**

| Characteristic and Statistic | | HCC  (n = 60) | Controls with CHB or Cirrhosis  (n = 60) | Healthy Controls  (n = 30) | *P* Value* |
| --- | --- | --- | --- | --- | --- |
| Age (year), median (IQR) | | 55 (49-62) | 49 (38-56) | 42 (37-49) | < 0.001 |
| Gender | |  |  |  |  |
|  | male (%) | 46 (76.7) | 37 (61.7) | 17 (56.7) | 0.094 |
|  | female (%) | 14 (23.3) | 23 (38.3) | 13 (43.3) |  |
| Etiology | |  |  | NA | 0.449 |
|  | HBV (%) | 56 (93.3) | 52 (86.7) |  |  |
|  | Alcohol (%) | 3 (5) | 5 (8.3) |  |  |
|  | NAFLD (%) | 0 (0) | 2 (3.3) |  |  |
|  | Other (%) | 1 (1.7) | 1 (1.7) |  |  |
| Cirrhosis (%) | | 54 (90) | 29 (48.3) | NA | < 0.001 |
| AFP (ng/mL) | |  |  |  |  |
|  | Median (IQR) | 19.3 (8.7-574.6) | 4.6 (2.9-9.6) | 2.4 (1.8-2.7) | < 0.001 |
|  | < 20 (%) | 31 (51.7) | 51 (85) | 30 (100) | < 0.001 |
|  | 20 - 400 (%) | 13 (21.7) | 8 (13.3) | 0 |  |
|  | > 400 (%) | 16 (26.6) | 1 (1.7) | 0 |  |
| BCLC stage | |  | NA | NA | NA |
|  | 0 (%) | 1 (1.7) |  |  |  |
|  | A (%) | 32 (53.3) |  |  |  |
|  | B (%) | 10 (16.7) |  |  |  |
|  | C (%) | 15 (25) |  |  |  |
|  | D (%) | 2 (3.3) |  |  |  |

*Statistical tests performed: Chi-square test of independence, Kruskal–Wallis test, and Wilcoxon rank-sum test.

**Table S6. Performance of different biomarker combinations in 150 plasma samples.**

| **CpG sites** | **Protein marker** | **AUC (95% CI)** | **Cut-off value** | **Sensitivity** | **Specificity** |
| --- | --- | --- | --- | --- | --- |
| cg14263942, cg12701184, cg14570307,  cg15457058, cg07689503, cg20172627 | AFP, DCP | 0.943 (0.893-0.974) | 0.310 | 88.3% | 92.2% |
| cg14263942, cg12701184, cg14570307,  cg15457058, cg07689503, cg20172627 | None | 0.918 (0.862-0.956) | 0.418 | 81.7% | 97.8% |
| cg14263942, cg12701184, cg14570307, cg15457058 | AFP, DCP | 0.938 (0.887-0.970) | 0.681 | 81.7% | 98.9% |
| cg14263942, cg12701184, cg14570307 cg07689503 | AFP, DCP | 0.938 (0.887-0.970) | 0.210 | 88.3% | 91.1% |
| cg14263942, cg12701184, cg14570307,  cg20172627 | AFP, DCP | 0.934 (0.882-0.968) | 0.499 | 81.7% | 96.7% |
| cg14263942, cg12701184, cg14570307 | AFP, DCP | 0.934 (0.882-0.968) | 0.631 | 81.7% | 98.9% |
| cg15457058, cg07689503, cg20172627 | AFP, DCP | 0.924 (0.869-0.961) | 0.456 | 80.0% | 94.4% |
| cg14263942, cg12701184, cg14570307 | None | 0.899 (0.839-0.942) | 0.411 | 81.7% | 96.7% |
| None | AFP, DCP | 0.874 (0.809-0.922) | 0.446 | 73.3% | 93.3% |

**Table S7. Limit of detection (LOD) of three methylation markers in HepaClear panel.**

| Template | Content of Huh7 cell DNA (pg/test) | Detection Rate | | |
| --- | --- | --- | --- | --- |
|  |  | cg14263942 (CDKL2) | cg12701184 (USP44) | cg14570307 (ZNF783) |
| 5%PC | 500 | 100% (20/20) | 100% (20/20) | 100% (20/20) |
| 1%PC | 100 | 100% (20/20) | 100% (20/20) | 100% (20/20) |
| 0.5%PC | 50 | 100% (20/20) | 100% (20/20) | 100% (20/20) |
| 0.25%PC | 25 | 85% (17/20) | 95% (19/20) | 80% (16/20) |
| NC | 0 | 0% (0/20) | 0% (0/20) | 0% (0/20) |

**Table S8. List of primers and probes for Taqman qMSP.**

| Gene | Note | Primer/Probe Sequence (5'-3') |
| --- | --- | --- |
| B2M | Forward Primer | GTAGGTTTGGGTAATTTTAAATAGTGGA |
|  | Reverse Primer | TTCTTTCAAAATATCATCCCCCAAT |
|  | Probe | TTCCTACAAATCTTCCCCCAAACACC |
| CDKL2 | Forward Primer | CGTTGGTCGCGGAGTTGTAA |
|  | Reverse Primer | GACTCGACTCGACCAATCAAAA |
|  | Probe | TAACGCTCTCGAAACGAA-MGB |
| USP44 | Forward Primer | GCGGTAGTTTTAGAGTGTTCGG |
|  | Reverse Primer | CAACTCGCCCCCTCCAAC |
|  | Probe | ATGTTGCGGTAAGGCGTAGCGG |
| ZNF783 | Forward Primer | TATTGGTTAATTCGGGAGGTTTC |
|  | Reverse Primer | CGCACCTATCGAACGTACACAA |
|  | Probe | AACCCGACGCACGCTATAAAAACGA |
| CDKN2A | Forward Primer | CGTTTTCGTTTTTAGTAGCGTTCGT |
|  | Reverse Primer | TAAAACCTTCGACTAACTAACTAACC |
|  | Probe | CGACCGCGACCCGAAATCGAA |
| LOXL3 | Forward Primer | CGTTTTTAATTTCGTTTGGAAGTTTTAG |
|  | Reverse Primer | ACCCGAATCCCGACGAAAA |
|  | Probe | TAAATCGATTTGCGTCGTAA-MGB |
| FOXE3 | Forward Primer | GCGTAAGTGGTAGAATAGTATTCGTT |
|  | Reverse Primer | TACCCTTACCCGAATTACCCGA |
|  | Probe | CGCGAAACACCTTAACGAAACAATCG |
| Chr2.25439110 | Forward Primer | TTGGGGTTCGTAGGTTTTAGGC |
|  | Reverse Primer | CTCGAAAAACCCGACTCGAAA |
|  | Probe | TTTCGTTTTAACGTATGCGCGCGTAT |
| TRIL | Forward Primer | TGGTTATGAAGTTGTCGTCGAGG |
|  | Reverse Primer | GCGTAATACCCAAAACCAACTCG |
|  | Probe | TAACCGAACCCCCACGACGTACTC |
| MTHFD2 | Forward Primer | TAGTATTTTAGTTAGAGGCGGTCG |
|  | Reverse Primer | ATAACGTACGAAACGCGAAAACATC |
|  | Probe | TTTACGCGAAACCTTCCGACAACGC |
| C5orf49 | Forward Primer | TCGCGTTTAGTTCGTTGGGC |
|  | Reverse Primer | CGAAACAACAAAACGCTCTTTCTAC |
|  | Probe | TTTCGGTTTTTCGCGATTTT-MGB |
